# Sex Differences in Disease Profiles, Management, and Outcomes Among People with Atrial Fibrillation After Ischemic Stroke: Aggregated and Individual Participant Data Meta-Analyses

**DOI:** 10.1089/whr.2020.0029

**Published:** 2020-06-30

**Authors:** Xia Wang, Hoang T. Phan, Jingwei Li, Mathew J. Reeves, Amanda G. Thrift, Dominique A. Cadilhac, Jonathan Sturm, Vemmos Konstantinos, Priya Parmar, Rita Krishnamurthi, Suzanne Barker-Collo, Valery Feigin, Norberto L. Cabral, Antonio Carolei, Carmine Marini, Simona Sacco, Manuel Correia, Peter Appelros, Janika Kõrv, Riina Vibo, Sook Ching Yang, Cheryl Carcel, Mark Woodward, Else Charlotte Sandset, Craig Anderson, Seana Gall

**Affiliations:** ^1^ The George Institute for Global Health, Faculty of Medicine, University of New South Wales, Sydney, Australia.; ^2^ Menzies Institute for Medical Research Tasmania, University of Tasmania, Hobart, Australia.; ^3^ Department of Health Management and Health Economics, Pham Ngoc Thach University of Medicine, Ho Chi Minh City, Vietnam.; ^4^ Department of Cardiology, People's Liberation Army General Hospital, Beijing, China.; ^5^ Department of Cardiology, Xinqiao Hospital, Army Military Medical University, Chongqing, China.; ^6^ Department of Epidemiology and Biostatistics, Michigan State University, East Lansing, Michigan, USA.; ^7^ Department of Medicine, School of Clinical Sciences at Monash Health, Monash University, Clayton, Australia.; ^8^ Faculty of Health and Medicine, University of Newcastle, Newcastle, Australia.; ^9^ Department of Clinical Therapeutics, Alexandra Hospital, National and Kapodistrian University of Athens, Athens, Greece.; ^10^ National Institute for Stroke and Applied Neurosciences, School of Public Health and Psychosocial Studies, Auckland University of Technology, Auckland, New Zealand.; ^11^ School of Psychology, University of Auckland, Auckland, New Zealand.; ^12^ Clinica Neurológica de Joinville, Joinville Stroke Registry, University of Joinville Region-Univille, Joinville, Brazil.; ^13^ Department of Biotechnological and Applied Clinical Sciences, Neurological Institute, University of L'Aquila, Italy.; ^14^ Department of Life, Health, and Environmental Sciences, University of L'Aquila, L'Aquila, Italy.; ^15^ InstitutodeCiênciasBiomédicasdeAbelSalazar, UniversidadedoPorto, Porto, Portugal.; ^16^ Department of Neurology, Faculty of Medicine and Health, Örebro University, Örebro, Sweden.; ^17^ Department of Neurology and Neurosurgery, Institute of Clinical Medicine, University of Tartu, Tartu, Estonia.; ^18^ Department of Cardiology, The Royal Infirmary of Edinburgh, Edinburgh, United Kingdom.; ^19^ Department of Neurology, Royal Prince Alfred Hospital, the University of Sydney, Sydney, Australia.; ^20^ The George Institute for Global Health, University of Oxford, Oxford, United Kingdom.; ^21^ Department of Neurology, Oslo University Hospital, Oslo, Norway.; ^22^ The George Institute China at Peking University Health Science Centre, Beijing, PR China.

**Keywords:** atrial fibrillation, ischemic stroke, sex differences

## Abstract

***Objectives:*** To examine sex differences in disease profiles, management, and survival at 1 and 5 years after ischemic stroke (IS) among people with atrial fibrillation (AF).

***Methods:*** We performed a systematic literature search of reports of AF at IS onset according to sex. We undertook an individual participant data meta-analysis (IPDMA) of nine population-based stroke incidence studies conducted in Australasia, Europe, and South America (1993–2014). Poisson regression was used to estimate women:men mortality rate ratios (MRRs). Study-specific MRRs were combined using random effects meta-analysis.

***Results:*** In our meta-analysis based on aggregated data from 101 studies, the pooled AF prevalence was 23% (95% confidence interval [CI]: 22%–25%) in women and 17% (15%–18%) in men. Our IPDMA is of 1,862 IS-AF cases, with women (79.2 ± 9.1, years) being older than men (76.5 ± 9.5, years). Crude pooled mortality rate was greater for women than for men (1-year MRR 1.24; 1.01–1.51; 5-year 1.12; 1.03–1.22). However, the sex difference was greatly attenuated after accounting for age, prestroke function, and stroke severity (1-year 1.09; 0.97–1.22; 5-year 0.98; 0.84–1.16). Women were less likely to have anticoagulant prescription at discharge (odds ratio [OR] 0.94; 95% CI: 0.89–0.98) than men when pooling IPDMA and aggregated data.

***Conclusions:*** AF was more prevalent after IS among women than among men. Among IS-AF cases, women were less likely to receive anticoagulant agents at discharge; however, greater mortality rate in women was mostly attributable to prestroke factors. Further information needs to be collected in population-based studies to understand the reasons for lower treatment of AF in women.

## Background

Worldwide, the number of men having atrial fibrillation (AF) is nearly twice that of women.^1,2^ However, women tend to have more severe AF symptoms and are at a higher risk of death and cardiovascular disease.^1,2^ In a comprehensive meta-analysis^3^ of 30 studies with 4,371,714 participants, AF was associated with a larger relative risk (RR) of stroke in women than in men (1.47, 95% confidence interval [CI]: 1.18–1.83). This might be explained by the presence of more comorbidities in women with AF than in men.^3^ Therefore, factors such as hypertension, older age, larger atrial dimensions, valvular disease, and cardiovascular remodeling have been suggested as plausible mechanisms leading to an apparent greater risk of stroke in women.^4^

Similarly, significant disparities in the burden of stroke between men and women have also been identified by the Global Burden of Disease (GBD) 2013 Study, with men having consistently greater incidence of ischemic stroke (IS) than women.^5^ However, the proportion of stroke-related deaths was greater in women than in men. In the INternational STRoke oUtComes sTudy (INSTRUCT), which included 16,957 participants from 13 population-based incidence studies, the greater mortality rate in women was largely attributable to age, but other important factors including stroke severity, presence of AF, and prestroke dependency also contributed to this disparity.^6^ These findings highlight the importance of investigating differences between women and men in the management and outcomes after stroke among participants with IS and AF.

Recently, the GBD 2016 investigators emphasized the importance of quantifying the attributable burden of AF for stroke.^7^ Indeed, in a large-scale registry study of 10,528 participants with IS in Canada, AF has been associated with an increased risk of death and severe disability. Older age and increased stroke severity were found to explain most of the association between AF and outcomes. However, observational evidence on how sex modifies the association between AF and outcomes is scarce.^8–11^ In this study, we performed a systematic review of existing evidence and meta-analysis using individual participant data (IPD) of nine studies from the INSTRUCT.^11^ Our aims were to outline the evidence for sex differences in disease profiles, management factors, and outcomes in participants with IS and AF.

## Methods

### Systematic review/meta-analysis of aggregated data of published studies

We included all studies in which AF was reported at the time of IS presentation, and were stratified according to sex. We also included participants aged at least 18 years, of any race with a clinical or imaging (computed tomography or magnetic resonance imaging) diagnosis of first-ever or recurrent IS. There were no language restrictions.

A comprehensive search strategy (in the Supplementary Data)—developed in consultation with a university librarian, neurologists, and epidemiologists—was used to address the unique features and indexing of each of the two electronic databases (MEDLINE and Embase), which were searched from inception to December 20, 2018. The systematic review was reported following Meta-analysis Of Observational Studies in Epidemiology guidelines.^12^ In addition to searching for original studies, the reference lists of any relevant reviews appearing in their reports were examined. Reference screening, data extraction, and quality assessment using Newcastle-Ottawa scale (NOS)^13^ were performed by J.L. and S.C.Y. Disagreements were resolved by a third author (X.W.).

The main outcomes of interest were the sex differences in the proportion of AF among participants with IS; also of interest were the stroke severity and functional outcomes (assessed by modified Rankin scale [mRS], Barthel Index (BI), or recurrent stroke) in participants with IS and AF.

The data were pooled using random effects models where data were available. The degree of heterogeneity was calculated using the *I^2^*-index. We also used meta-regression to assess whether sex differences in AF prevalence were modified by age.

### Meta-analysis of IPD

The INSTRUCT is an IPD database of long-term outcomes after first-ever stroke comprising 13 “gold standard” population-based stroke incidence studies,^14,15^ which have greater internal validity and less selection bias than hospital-based studies.^16^ The details of the design of INSTRUCT have been described elsewhere.^6,17,18^

This analysis focused on participants with IS and AF in the INSTRUCT, and included nine studies whose investigators have agreed to participate (Supplementary Table S1).^19–27^ These studies were conducted in Australia, New Zealand, Brazil, Greece, Sweden, Portugal, Italy, and Estonia between 1993 and 2014. This study was approved by the Tasmanian Health and Medical Human Research Ethics Committee (H0014861). All of the participating studies had signed informed consent and approval from their respective local ethics committees.

### Outcome measurement

Outcomes of stroke included all-cause mortality and functional outcomes up to 5 years after stroke. The details of study outcomes of the INSTRUCT have been described elsewhere.^6,18^ Measures of mortality rate at 1 year were available among all nine studies, whereas those at 5 years were available among 5five of nine studies. Mortality rate was obtained from national death registries (the studies from Melbourne, Perth, Orebro, and Tartu) or the combination of hospital records, death certificates, or participant follow-ups (remaining studies: Joinville, Arcadia, Porto, Auckland, L'Aquila).

Six studies had functional outcomes measurement assessed by the mRS (score range 0–5) or BI (score range 0–20) at 1 year, whereas only three studies had 5-year functional outcomes after stroke. The mRS or BI scores were assessed by research nurses or attending physicians face to face or by telephone (Supplementary Table S1). Poorer functional outcome was defined as mRS >2 or BI <20 at 1 or 5 years after stroke, mRS was used if both were available.

### Study factors

The presence of AF was self-reported by participants in two studies and was confirmed by ECG or medical record in the remaining seven studies (Supplementary Table S1). The methods for ascertainment of other study factors are described elsewhere.^6^ For each study, a wide range of factors that might contribute to sex differences were recorded.^28^ These were (1) sociodemographics, (2) prestroke health (dependence, comorbidities, and health behaviors), (3) stroke-related factors (stroke severity and year of stroke occurrence), and (4) treatment and management.

### Statistical analysis

Because of the inconsistency of covariate measurements between studies from different populations, we used the two-stage method of analysis proposed for IPD meta-analysis^29^ using the same approach demonstrated in previous publications from INSTRUCT, including for mortality^6^ and functional outcome.^18^

The first stage involved building study-specific crude and adjusted models to estimate women:men mortality rate ratio (MRR) or RR of having poorer functional outcomes for women compared with men. For mortality outcome, we used Poisson regression with the logarithm of the number of person-years at risk of dying within that period entered as an offset.^30^ For functional outcome, multivariable log-binomial regression was performed.

Within each study, we assessed the confounding role^31^ of covariates in the association between sex and each outcome. The following rules were applied to determine the confounders in the study-specific multivariable models: (1) the covariate was associated with mortality, (2) the covariate was associated with sex, and (3) the inclusion of the covariate in a model with only sex changed the magnitude of the sex coefficient by ≥10%.^31^ Adjustment was done for each variable separately and then for all confounding factors in multivariable analyses, but with age, stroke severity, and prestroke function (where available) were forced into a final fully adjusted model. Within each study, statistical interactions were assessed by a test of statistical significance of a sex × covariate product term. Covariates chosen for the test include age, stroke severity, and prestroke function, and all other significant confounding factors. To further examine the robustness of our findings, we also tested interaction effects between sex and participant-level covariates including stroke type, age at stroke onset, and the year of stroke occurrence.

For the second stage of the analysis, unadjusted and adjusted study-specific estimates were pooled in separate random effects meta-analyses, so that the pooled values could be compared to determine the effect of adjustment. Heterogeneity was evaluated using Q statistics and *I*^2^ statistics. Meta-regression was used to identify the sources of statistically significant heterogeneity among study**-**level characteristics.

### Pooling meta-analysis of aggregated data and meta-analysis of IPD

Where possible, IPD meta-analysis and meta-analysis based on aggregated data from the literature were pooled using random effects models and odds ratios (ORs) were reported. Two-sided *p*-values of ≤0.05 were deemed statistically significant. All statistical analyses used Stata, version 12.1.

## Results

### Systematic review/meta-analysis of aggregated data of published studies

Of 15,127 references obtained after execution of the search strategy, 3,057 remained after screening titles and abstracts for relevance (Supplementary Fig. S1). One hundred and one studies (*n* = 2,298,873 participants) that satisfied the eligibility criteria were included in the review (Supplementary Table S2). Four studies^32–35^ were defined as low quality with scores <5 using NOS. Begg's regression tests identified no evidence of publication bias (*p* = 0.07).

The pooled proportion of AF in participants with IS was 23% (95% CI: 22%–25%) in women and 17% (15%–18%) in men, the *I*^2^ was 98.6% and 96.8%, respectively (Supplementary Figs. S2 and S3). Among participants with IS and AF, women were much older than men. However, meta-regression demonstrated that sex differences in AF prevalence after stroke were consistent with increasing age (*p* = 0.243, Supplementary Fig. S4).

In five out of six studies with data on stroke severity by sex, women with AF had more severe IS than men at IS onset, as assessed by National Institutes of Health Stroke Scale (NIHSS), mRS or CHADS_2_ score (Supplementary Table S3). Antiplatelet therapy by sex at IS onset or discharge was reported in a lower proportion among women with AF than among men (9 of 11 studies; Supplementary Table S4). More women with AF than men had a history of hypertension at IS onset in all five studies, whereas fewer women with AF reported a history of diabetes than men in four of five of the studies (Supplementary Table S5). Women with AF tended to have poorer outcomes than men after IS (6 of 6 studies; Supplementary Table S6).

### Meta-analysis of IPD using the INSTRUCT

**FIG. 1.**
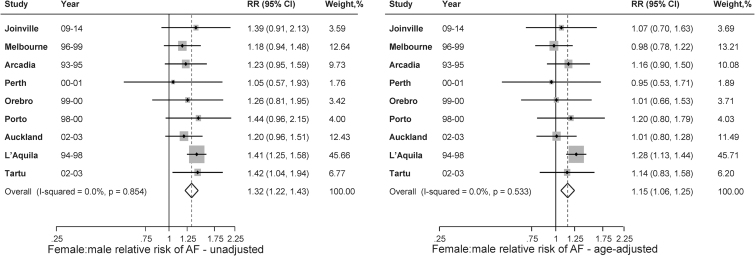
The pooled proportion of AF for women and men in the included studies of individual patient data meta-analysis. AF, atrial fibrillation.

There were 8,645 participants from the nine studies with a mean age of 72.6 years and 51.8% (4,480) being women (Supplementary Table S7). The proportion of AF varied across studies ranging from 6.2% to 42.3% in women and from 4.4% to 34.4% in men (Supplementary Table S7). The pooled proportion of AF was 26.6% in women and 20.8% in men. The prevalence was higher in women than in men, even after allowing the differences explained by age (RR 1.15, 95% CI: 1.06–1.25) (Fig. 1).

Table 1 shows the baseline characteristics of participants with IS who have AF (*n* = 1,862), further stratified by sex in each study (Supplementary Tables 8 and 9). Women were older, more likely to be living in institutions, and more functionally dependent before stroke than men. More women than men had a history of peripheral vascular disease or transient ischemic attack (TIA), whereas men were more often smokers and drinkers. Women suffered more severe stroke than men.

**Table 1.** Characteristics of Nine Included Cohort Studies in Patients with Ischemic Stroke and Atrial Fibrillation, by Sex

Characteristic	Women, *n* (%)	Men, *n* (%)	*p*
Sociodemographics			
Mean (SD) Age (nine studies)	79.2 (9.1)	76.5 (9.5)	**<0.001**
Marital status (three studies)			
Single/widowed	54 (37.5)	121 (65.8)	**<0.001**
Married	88 (61.1)	60 (32.6)	
Unknown	2 (1.4)	3 (1.6)	
Education level (four studies)			
≤Grade 12	144 (53.7)	213 (58.4)	0.352
>Grade 12	86 (32.1)	98 (26.9)	
Unknown	38 (14.2)	54 (14.8)	
Social class (four studies)			
Professional	61 (24.7)	50 (14.9)	**<0.001**
Nonmanual	55 (22.3)	106 (31.6)	
Manual	94 (38.1)	78 (23.2)	
Unknown	37 (15.0)	102 (30.4)	
Prestroke health			
In an institution (three studies)			
Yes	16 (11.4)	28 (15.0)	0.607
No	123 (87.9)	157 (84.0)	
Unknown	1 (0.7)	2 (1.1)	
Modified Rankin Score (mRS; four studies)			
0–2	138 (87.3)	184 (81.1)	0.253
3–5	10 (6.3)	23 (10.1)	
Unknown	10 (6.3)	20 (8.8)	
Barthel Index score (BI; three studies)			
20	74 (52.9)	83 (44.4)	0.316
<20	23 (16.4)	36 (19.3)	
Unknown	43 (30.7)	68 (36.4)	
Mean (SD) mRS	0.7 (1.0)	1.1 (1.1)	**0.002**
Mean (SD) BI	18.6 (3.7)	18.8 (3.1)	0.338
Medical history			
Hypertension (nine studies)			
Yes	457 (60.0)	712 (64.7)	0.085
No	295 (38.7)	371 (33.7)	
Unknown	10 (1.3)	17 (1.6)	
Ischemic heart disease (nine studies)			
Yes	248 (32.6)	324 (29.5)	0.210
No	509 (66.8)	763 (69.4)	
Unknown	5 (0.7)	13 (1.2)	
Peripheral vascular disease (five studies)			
Yes	92 (16.2)	123 (14.9)	**0.001**
No	471 (82.9)	670 (80.9)	
Unknown	5 (0.9)	35 (4.2)	
Transient ischemic attack (eight studies)			
Yes	82 (12.4)	22 (2.3)	**0.042**
No	566 (85.9)	865 (89.0)	
Unknown	11 (1.7)	22 (2.3)	
Diabetes (four studies)			
Yes	49 (23.0)	50 (19.5)	0.648
No	163 (76.5)	205 (80.1)	
Unknown	1 (0.5)	1 (0.4)	
Dementia (three studies)			
Yes	13 (5.7)	24 (8.1)	0.520
No	192 (84.2)	241 (80.9)	
Unknown	23 (10.1)	33 (11.1)	
Smoking (eight studies)			
Never	319 (43.9)	804 (78.5)	**<0.001**
Current	145 (19.9)	92 (9.0)	
Former	209 (28.8)	39 (3.8)	
Unknown	54 (7.4)	89 (9.7)	
Alcohol use (six studies)			
Nondrinkers	134 (37.0)	269 (59.4)	**<0.001**
Not heavy drinkers	70 (19.3)	57 (12.6)	
Heavy drinkers	90 (25.1)	51 (11.3)	
Ex-drinkers	22 (6.1)	16 (3.5)	
Unknown	45 (12.4)	60 (13.3)	
Stroke-related factors			
Hospital admission (nine studies)			
Yes	737 (96.7)	1,064 (96.7)	0.992
No	25 (3.3)	36 (3.3)	
Time to hospital arrival (six studies)			
≤4.5 hours	61 (18.9)	121 (26.1)	0.050
>4.5–24 hours	112 (34.7)	157 (33.9)	
>24 hours	24 (7.4)	21 (4.5)	
Unknown	126 (39.0)	164 (35.4)	
Stroke severity			
Mean (SD) NIHSS score (five studies)	9.1 (8.1)	10.8 (8.5)	**0.029**
Mean (SD) GCS score, reversed (two studies)	3.3 (2.9)	3.6 (3.1)	0.402
Loss of consciousness (five studies)			
Yes	179 (27.8)	272 (29.7)	0.611
No	434 (67.3)	595 (64.9)	
Unknown	32 (5.0)	50 (5.5)	
Medications at discharge			
Antiplatelet agents (three studies)			
Yes	108 (44.3)	176 (53.3)	**0.040**
No	136 (55.7)	152 (46.1)	
Unknown	0 (0)	2 (0.6)	
Anticoagulant agent (three studies)			
Yes	71 (32.7)	71 (25.7)	0.146
No	143 (65.9)	197 (71.4)	
Unknown	3 (1.4)	8 (2.9)	

Bold denotes statistically significant results.

BI, Barthel Index; GCS, Glasgow Coma Scale; mRS, modified Rankin scale; NIHSS, National Institutes of Health Stroke Scale; SD, standard deviation.

**FIG. 2.**
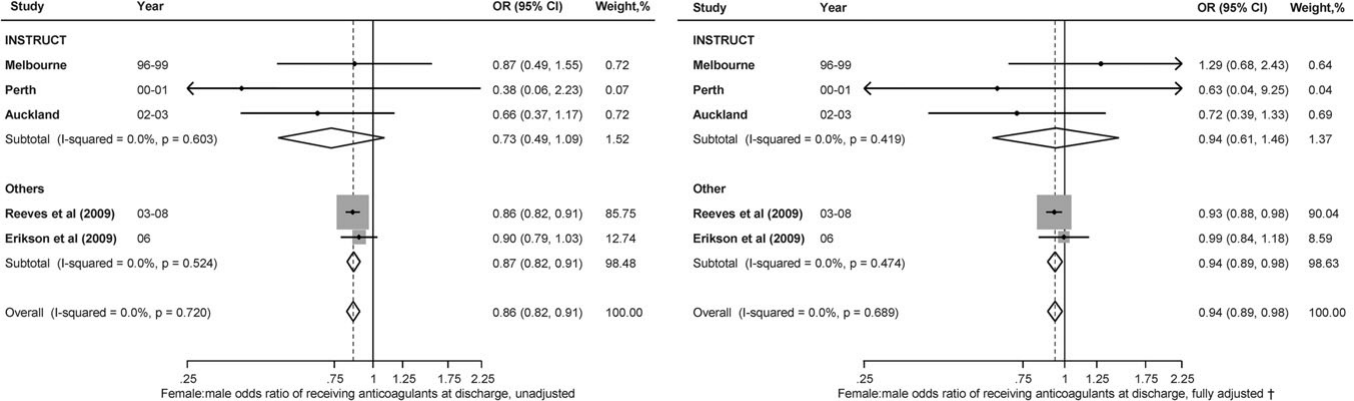
Sex differences in receiving anticoagulant agents at discharge among those with AF and ischemic stroke (*n* = 45,782). CI, confidence interval; OR, odds ratio.

Among participants with IS who have AF, women tended to have more severe stroke (NIHSS score, NIHSS >7) than men (Supplementary Fig. S5), although this difference did not reach statistical significance. Women were less likely to have history of ischemic heart disease (0.87, 0.76–0.99). There was no significant sex difference in comorbidities including TIA (RR 0.78, 95% CI: 0.58–1.05), hypertension (RR 1.07, 95% CI: 0.99–1.17), and diabetes (RR 1.05, 95% CI: 0.73–1.49) (Supplementary Fig. S6). There were no significant differences in receiving anticoagulant (RR 0.94, 95% CI: 0.61–1.46; Fig. 2), antihypertensive, and antiplatelet agents (Supplementary Fig. S7).

**FIG. 3.**
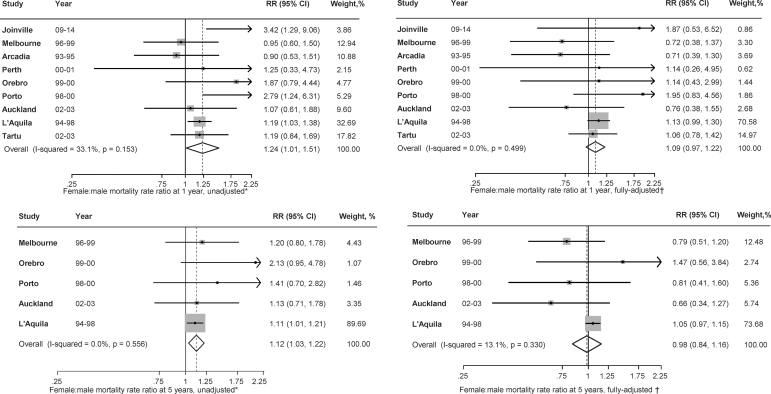
Sex differences in mortality rate at 1 year (*n* = 1,862) and 5 years (*n* = 616) after ischemic stroke in those with AF. RR, relative risk. *Estimates were from univariate analysis; ^†^Estimates were from multivariable analysis.

The survival rate using pooled IPD for people with AF and IS was 51.7% in women and 58.9% in men at 1 year (9 studies) and 31.9% in women and 36.8% in men at 5 years (5 studies; Supplementary Table S10). Women were 24% more likely than men to have died within 1 year in crude analyses (RR 1.24, 95% CI: 1.01–1.51), without evidence of heterogeneity (*I*^2^ = 34; Q = 16.3; *p* = 0.146; Fig. 3 and Supplementary Tables S11 and S12). However, after adjustment for confounders including age, the strength of the association was attenuated and the difference in mortality rate was no longer statistically significant (RR 1.09, 0.97–1.22). In crude analyses of 5-year mortality rate, women had a trend to be 12% more likely than men to die after stroke (MRR 1.12, 95% CI: 1.03–1.22; Fig. 3). After adjusting for covariates including age, severity and prestroke disability, there was no difference in survival between men and women (MRR 0.98, 95% CI: 0.84–1.16; Fig. 3). When the models were adjusted for age, severity, and prestroke dependency separately, we found that the coefficient of the sex difference in RR was attenuated most with adjustment for age (Supplementary Table S13).

**FIG. 4.**
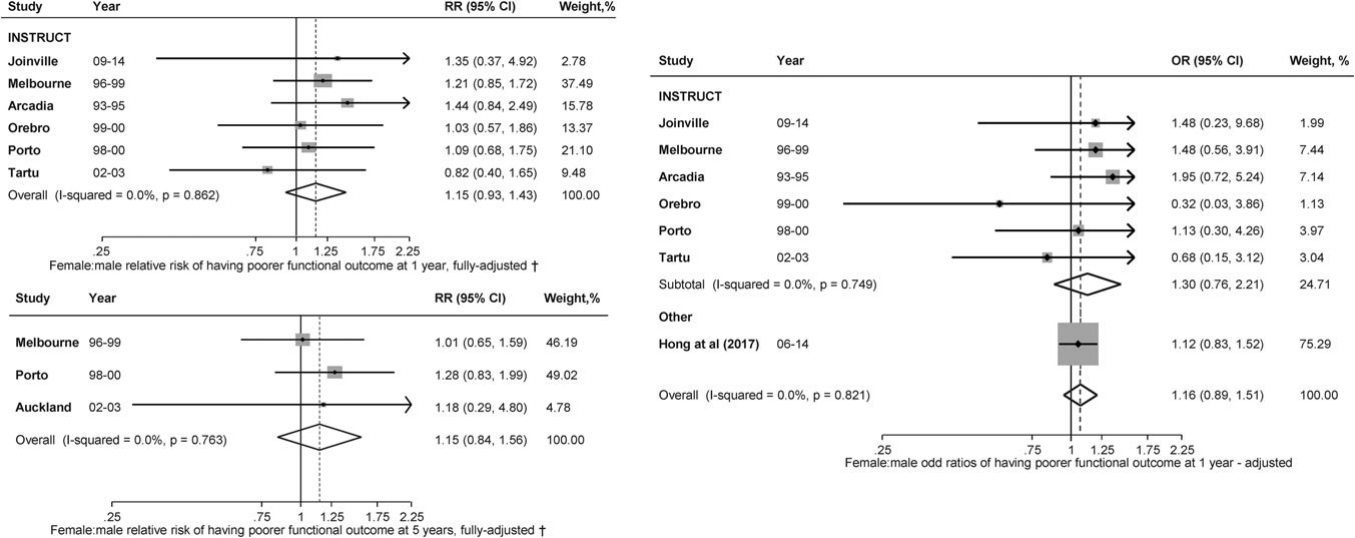
Sex differences in functional outcome (modified Rankin Scale >2 or Barthel Index <20) at 1 year (*n* = 1,681) and 5 years (*n* = 551) after ischemic stroke in those with AF. ^†^Estimates were from multivariable analysis.

The proportion of poor functional outcome using the pooled IPD among people with IS and AF was 41.3% (women) and 31.5% (men) at 1 year (6 studies) and 35.5% (women) and 23.4% (men) at 5 years (3 studies; Table 1). At 1 year, women were 61% more likely than men to have poor functional outcomes in crude analyses (RR 1.61, 95% CI: 1.27–2.04) without evidence of heterogeneity (*I*^2^ = 0%; *p* = 0.642). However, after adjusting for confounders including age, the magnitude of the difference was attenuated (RR 1.15, 95% CI: 0.93–1.43) (Fig. 4). At 5 years, women were not significantly different from men in terms of having poor functional outcomes in crude (RR 1.12, 95% CI: 1.03–1.22) and adjusted (RR 0.98, 95% CI: 0.84–1.16) analyses. When the models were adjusted for age, severity, and prestroke dependency separately, we found that the coefficient of the sex difference in RR was attenuated most with adjustment for age (Supplementary Tables S14–S17).

### Pooled meta-analysis of aggregated data and IPDMA

Women were less likely to receive anticoagulant agents at discharge (age-adjusted pooled OR women vs. men 0.94, 95% CI: 0.89–0.98, Fig. 2). Women were 23% more likely than men to have died within 1 year in crude analyses (OR 1.23, 95% CI: 1.00–1.51), without evidence of heterogeneity (*I*^2^ = 34; Q = 16.3; *p* = 0.146). However, after adjustment for confounders including age, the strength of the association was attenuated and the difference in mortality rate was no longer statistically significant (OR 1.09, 0.97–1.22) (Fig. 3).

## Discussion

In this systematic review and meta-analysis, we found that AF was more prevalent after IS among women than among men. Among participants with IS who have AF, women were older than men, they tend to have more severe strokes, more often have a history of hypertension and diabetes, but less often had ischemic heart disease and TIA. Women were less likely to be prescribed anticoagulant agents at discharge from hospital. The greater mortality rate and poorer functional outcome after stroke in women were mostly attributable to their differences in clinical profile, including advanced age, greater stroke severity, and prestroke functional limitations.

Our IPD data provide further evidence that age was the most important contributor to the sex difference in poor prognosis among participants with IS who have AF.^6,36,37^ This could be explained by several reasons. First, given that AF increases with age and that women have greater life expectancy, we would expect an increasing proportion of women with AF as the population ages.^38^ Furthermore, women are more likely than men to experience AF-related symptoms, to have significantly higher heart rates during AF, and a less favorable response to treatment.^39^ Piccini et al. reported that women have higher stroke risk despite equal anticoagulant use.^40^ It has also been observed that women with AF have more advanced atrial fibrosis than men. These sex differences are more pronounced with a history of IS, suggesting that sex may play a role in fibrotic remodeling of the left atrium and subsequent stroke.^41,42^ An added factor^43^ to consider, which is rarely included in population-level studies, is hormone replacement therapy (HRT). HRT and particularly conjugate equine estrogens have been linked to increased risk of AF as well as stroke and, of course, HRT use also increases with age so could potentially contribute to the age effect.

With respect to evidence-based medications for secondary prevention, out data show women were less likely to receive anticoagulation therapy at discharge. However, evidence from the literature was not consistent for either Western^44–47^ or Asian countries.^11,48,49^ Use of warfarin at discharge was less in women than in men in Get With The Guidelines-Stroke program^47^ and the Swedish Risk-Stroke hospital-based registry,^44^ whereas in studies from the Canadian stroke registry^45^ and Medicare participants in Michigan, there were no sex differences.^46^ This might be due to the fact that current evidence on appropriateness of anticoagulation is controversial in people with acute IS who have AF. The American Heart Association/American Stroke Association does not recommend anticoagulation for treatment of acute IS because of lack of benefit.^50–52^ Whereas evidence from studies with AF participants suggested that women, especially those aged ≥75 years, most benefit from anticoagulation therapy.^53,54^ Similarly, it has been reported that women with acute coronary syndrome were less likely to receive evidence-based acute treatment and medical therapies for secondary prevention.^55^ However, the reasons for these disparities were largely unknown.^56^ Potential explanations include sex differences in eligibility for therapy, clinical contraindications, and other clinical factors.^57^

Women with IS who have AF tended to have more severe strokes than men, which could be explained by older age and higher vascular burden. One large population-based study^36^ that included ∼40,000 patients provided evidence that the major risk factor associated with stroke was advanced age, but that female sex was the major risk factor among participants with AF. Older participants (≥75 years) were the most vulnerable population. This group has the highest rates of AF and the greatest risk of stroke. Women tend to have much higher vascular burden, reflected as a higher mean CHADS_2_ score and, therefore, they more often suffer larger strokes associated with severe neurological effects depending on more large vessel occlusions.

Our study has several strengths. This is the first IPD meta-analysis of population-based studies to explore the magnitude of sex difference in both short- and long-term mortality in participants with IS who have AF. The data come from high-quality and generalizable studies free of the limitations of hospital-based or convenience samples. We have synthesized all the currently available evidence by pooling IPD and meta-analysis results. However, several limitations need to be noted. There is considerable heterogeneity in AF prevalence, which might be due to multiple factors such as time, self-reported versus diagnosed, and ethnic differences. Second, some potential confounding factors were not measured including hormonal, social, and some demographic factors, particularly race or ethnicity. Third, AF might be underestimated in this study. It was self-reported in two included studies and also there could be underascertainment when relying on hospital records (as absence in the medical record does not necessarily equate to absence of the risk factor). We had no information whether the diagnosis of AF was concomitant to IS, previously, or newly diagnosed. Furthermore, the diagnosis of IS was not always imaging based. A merely clinical-based diagnosis of IS could be less accurate. Both first-ever and recurrent IS were included, and prognosis of recurrent IS can be different, which might impact the generalizability of the study. In addition, some of the included studies in the IPD were conducted in the late 1990s and in that the populations were largely Caucasian. Diagnostic techniques for AF have improved from 1990 to nowadays, as well as the management of AF. This might have impacted prognosis of IS over time. However, we conducted a systematic review to synthesize the most updated published data and pooled the aggregated data with IPD where possible to make sure the data were relevant to current clinical practice.

In conclusion, the poorer outcome in women with IS and AF was mostly attributable to prestroke factors. More reliable evidence is needed to understand sex disparities in evidence-based care for secondary prevention especially anticoagulation. To better understand the clinical differences observed in presentation, treatment, and outcome of IS and AF in men and women, we suggest future population-based studies collect information on a range of reasons including socioeconomic factors, barriers to access medications, and biomarkers for biological differences that might explain these differences.

## Supplementary Material

Supplemental data

Supplemental data

Supplemental data

Supplemental data

Supplemental data

Supplemental data

Supplemental data

Supplemental data

## Notes

**Cite this article as:** Wang X, Phan HT, Li J, Reeves MJ, Thrift AG, Cadilhac DA, Sturm J, Konstantinos V, Parmar P, Krishnamurthi R, Barker-Collo S, Feigin V, Cabral NL, Carolei A, Marini C, Sacco S, Correia M, Appelros P, Kõrv J, Vibo R, Yang SC, Carcel C, Woodward M, Sandset EC, Anderson C, Gall S (2020) Sex differences in disease profiles, management, and outcomes among people with atrial fibrillation after ischemic stroke: aggregated and individual participant data meta-analyses, *Women's Health Report* 1:1, 190–202, DOI: 10.1089/whr.2020.0029.

## Abbreviations Used

AF: atrial fibrillation
BI: Barthel Index
CI: confidence interval
GBD: Global Burden of Disease
GCS: Glasgow Coma Scale
HRT: hormone replacement therapy
INSTRUCT: INternational STRoke oUtComes sTudy
IPD: individual participant data
IPDMA: individual participant data meta-analysis
IS: ischemic stroke
MRR: mortality rate ratio
mRS: modified Rankin scale
NIHSS: National Institutes of Health Stroke Scale
NOS: Newcastle-Ottawa scale
OR: odds ratio
RR: relative risk
SD: standard deviation
TIA: transient ischemic attack
