# Supplementary material for: Sex Differences in Disease Profiles, Management, and Outcomes Among People with Atrial Fibrillation After Ischemic Stroke: Aggregated and Individual Participant Data Meta-Analyses
Source: Womens Health Rep (New Rochelle). 2020 Jun 30;1(1):190–202. doi: 10.1089/whr.2020.0029 (PMC7784810; doi:10.1089/whr.2020.0029)
Supplement: Supplemental data [file Suppl_FigS1.pdf]

Supplementary Data

Search Strategy

MEDLINE

(1) exp STROKE/
(2) (stroke or poststroke or post-stroke).mp. [mp = title, abstract, heading word, drug trade name, original title, device manufacturer, drug manufacturer, device trade name, keyword, floating subheading word]
(3) Sex Factors/
(4) ((sex or gender) adj2 distribution).mp.
(5) ((sex or gender) adj2 difference*).mp.
(6) (gender-based or gender-related or gender differences or gender factors).mp.
(7) ((sex or gender) adj3 (analysis or factor$ or inequit$ or disparit$ or inequalit$ or difference$ or inter-act$)).mp.
(8) 1 or 2
(9) 3 or 4 or 5 or 6 or 7
(10) 8 and 9
(11) limit 10 to (humans and "all adult (19 plus years)")

Embase Classic and Embase

(1) exp STROKE/
(2) (stroke or poststroke or post-stroke).mp. [mp = title, abstract, heading word, drug trade name, original title, device manufacturer, drug manufacturer, device trade name, keyword, floating subheading word]
(3) Sex Factors/
(4) ((sex or gender) adj2 distribution).mp.
(5) ((sex or gender) adj2 difference*).mp.
(6) (gender-based or gender-related or gender differences or gender factors).mp.
(7) ((sex or gender) adj3 (analysis or factor$ or inequit$ or disparit$ or inequalit$ or difference$ or inter-act$)).mp.
(8) 1 or 2
(9) 3 or 4 or 5 or 6 or 7
(10) 8 and 9
(11) limit 10 to (human and (adult <18 to 64 years> or aged <65+ years>))

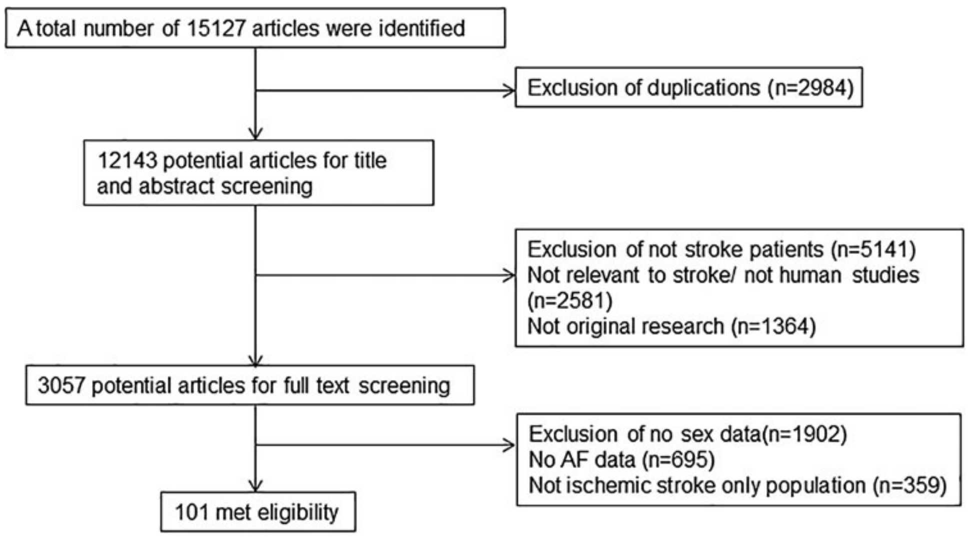

SUPPLEMENTARY FIG. S1. Literature search and selection process.
