# Supplementary material for: Sex Differences in Disease Profiles, Management, and Outcomes Among People with Atrial Fibrillation After Ischemic Stroke: Aggregated and Individual Participant Data Meta-Analyses
Source: Womens Health Rep (New Rochelle). 2020 Jun 30;1(1):190–202. doi: 10.1089/whr.2020.0029 (PMC7784810; doi:10.1089/whr.2020.0029)
Supplement: Supplemental data [file Suppl_TableS1-S17.pdf]

Supplementary Table S1. Atrial Fibrillation and Functional Outcome Measurement in Each Study

Study	Study year
Joinville, Brazil[S1]	A self-reported history of known AF was obtained from patients or their relatives by research nurses.<br>Functional outcomes were determined using mRS by research nurses using telephone.
Melbourne, Australia[S2]	AF was defined as either a known history or current presentation confirmed on ECG.<br>Functional outcomes at 1 and 5 years were determined using BI by research nurses in a face-to-face interview.
Arcadia, Greece[S3]	AF was diagnosed by ECG for hospitalized patients; for those not hospitalized, ≥1 ECG with documentation of AF in the last year before the event was required.<br>Functional outcomes at 1 year were determined using mRS by attending physicians face to face.
Perth, Australia[S4]	The presence of AF needed to be confirmed by an ECG within 1 month of the onset of stroke.<br>Patients not known to be deceased were sent a letter of invitation, which was followed by a telephone call or home visit. Functional outcomes at 1 year were determined using mRS. Those who agreed to participate were assessed at their usual place of residence by the study nurse.
Orebro, Sweden[S5]	AF was self reported by patients.<br>The functional outcomes at 1 year were measured using mRS during consultation visits or telephone interviews.
Porto, Portugal[S6]	The presence of AF was confirmed by ECG<br>Functional outcomes at 1 and 7 years were determined using mRS by study neurologists. Patients who collaborated but were not willing to complete the consultation were contacted by phone, and for those unable to come to the hospital, home visits were scheduled. Functional outcome was measured using the mRS.
Auckland, New Zealand[S7]	A self-reported history of AF was obtained from patients or their relatives and then confirmed by medical records.<br>Face-to-face interviews occurred at participants' usual places of residence and included neuropsychological tests and assessment of functional outcome at 5 years (the mRS) after stroke.
L'Aquila, Italy[S8,S9]	AF was confirmed by a standard 12-lead ECG.<br>Functional outcome assessments were not available.
Tartu, Estonia[S10]	AF was confirmed by ECG<br>The mRS assessments were made by the study physician by telephone at 1 year and 4 years after stroke.

AF, atrial fibrillation; BI, Barthel Index; mRS, modified Rankin Scale.

Supplementary Table S2. Characteristics of Included Studies

Authors and year of publication	Country and city	Total case	Study type	Population	Female					Male				
					n	No. of AF	%AF	Age	NIHSS	*n*	No. of AF	%AF	Age (years)	NIHSS
Kent 2005[S11]	International	2,178	Randomized controlled trial	IS	988	195	19.7	66.5 (11.5)	13.0 (6.4)	1,190	213	17.9	65.4 (11.3)	13.0 (6.8)
Lasek-Bal 2014[S12]	Poland	511	Observational clinical cohort study	IS	304	101	33.2	74 (66–81)	7.1	207	41	19.8	70 (61–77)	5.63
Akanksha 2017[S13]	India	203	Population-based registry	IS&AF	83	34	41			120	71	59		
Yesilot 2011[S14]	Turkey, Istanbul	1,522	Hospital registry data	IS	751	252	33.6	65.1 (15.3)	Prestroke mRS3–5 11.5%	771	144	18.7	63.6 (14.6)	Prestroke mRS3–5 5.9%
Irie 2015[S15]	Fukuoka, Japan	6,236	Population-based registry	IS	2,398	701	29.2	73.3 (12.3)	4 (2–10)	3,838	968	25.2	68.3 (11.5)	4 (2–7)
Huang 2010[S16]	China	4,782	Population-based registry	IS	1,810	162	9	66.2 (11.8)		2,972	152	5	63.7 (11.8)	
Hametner 2017[S17]	International	8,028	Randomized controlled trial	IS	3,630	1,149	31.7	74 (65–80)	13 (8–18)	4,398	1,038	23.6	70 (60–77)	12 (8–17)
Hametner 2015[S18]	Germany	1,391	Observational clinical cohort study	IS	668	272	40.7	75.3 (12.1)	12 (7–17)	723	185	25.6	68.8 (12.7)	10 (6–15)
Gray 2007[S19]	International	1,268	Randomized controlled trial	IS	574	69	21.3	73.4 (10.7)	Scandinavian Stroke Scale 35 (16)	694	57	15.4	68.4 (10.8)	Scandinavian Stroke Scale 36 (18)
Nakamura 2016[S20]	Fukuoka, Japan	704	Population-based registry	IS&AF	298		42			406		58		
Munoz-Rivas 2015[S21]	Spain	423,475	Observational study using the Spanish National Hospital Database	IS	202,057	66,679	33			221,418	44,979	20.3		
Martin-Merino 2011[S22]	United Kingdom	2,953	Population-based registry	IS	1,455	233	16			1,498	188	12.6		
Kongbunkiat 2015[S23]	Thailand	25,319	Observational study using the Thailand national database system	IS/TIA&AF	14,699		58.1			10,620		41.9		
Giralt-Steinhauer 2012[S24]	Barcelona,Spain	439	Hospital registry data	IS/TIA&AF	270		61.50342			169		38.49658		
Kim 2015[S25]	Seoul, Republic of Korea	98	Hospital registry data	IS/TIA&AF	34		34.7			64		65.3		
Heuschmann 2004[S26]	Germany	1,3440	Population-based registry	IS	5,049	1,302	25.8	73.2 (13)		5,751	1,006	17.5	67.3 (12)	
Candelise 1991[S27]	Italy	211	Randomized controlled trial	IS&AF	126		59.71564			85		40.28436		
Kapral 2006[S28]	Toronto, Canada	586	Hospital registry data	IS	263	31	12	55		323	43	13	62	
Kapral 2009[S29]	Toronto, Canada	6,389	Population-based registry	IS	3,096	603.72	19.5			3,293	451.141	13.7		
Lin 2011[S30]	Sichuan, China	366	Hospital registry data	IS&AF	216		59.01639			150		40.98361		
Lisabeth 2009[S31]	Michigan, United States	461	Hospital registry data	IS/TIA	224	49	21.9			237	37	15.6		
Lisabeth 2004[S32]	Michigan, United States	2,715	Medicare population	IS/TIA	1,522	179.596	11.8			1,193	150.318	12.6		
Lorenzano 2013[S33]	International	45,079	Population-based registry	IS	18,777	5,603	29.8			25,219	5,372	21.3		
Marini, 2005[S9]	L'Aquila, Italy	3,530[a]	Population-based registry	IS	1,854	529	60.9	AGE IS NOT REPORTED		1,676	340	39.1	AGE IS NOT REPORTED	
Martinez-Sanchez 2011[S34]	Madrid, Spain	310	Observational clinical cohort study	IS	128	8	6.3			182	10	5.5		

(continued)

Supplementary Table S2. (Continued)

Authors and year of publication	Country and city	Total case	Study type	Population	Female					Male				
					n	No. of AF	%AF	Age	NIHSS	*n*	No. of AF	%AF	Age (years)	NIHSS
Mcdermott 2017[S35]	Texas, United States	757	Population-based brain attack surveillance	IS	391	68	17.4	72 (60,81)	5	366	45	12.3	65 (57,77)	4
Meseguer 2009[S36]	Paris, France	274	Hospital registry data	IS&thrombolysis	111	39	35.2	70.6 (16.1)	12 (7–17)	163	38	23.3	62.7 (13.9)	15 (9–19)
Mizrahi 2011[S37]	Israel	707	Hospital registry data	IS	303	79	26.1			404	47	11.6		
Nagaraja 2012[S38]	Detroit, United States	602	Hospital registry data	IS	320	51	15.9			282	34	12.1		
Mizrahi 2012[S39]	Israel	919	Observational clinical cohort study	IS	405	246	60.7	76.2 (7.9)		514	202	39.3	75.3 (8.0)	
Olsen and Andersen 2010[S40]	Demark	4,0155	National Stroke registry	IS	19,301	3,443	17.8	74.5		20,854	2,860	13.7	69.7	
Padjen 2013[S41]	Lille, France, and Belgrade, Serbia	734	Observational clinical cohort study	IS&thrombolysis	347	90	25.9			387	65	16.8		
Rodriguea-Campello 2016[S42]	Barcelona,Spain	388	Observational clinical cohort study	IS	140	24	17.1			248	31	12.5		
Silva 2010[S43]	Brazil	676	Hospital registry data	IS	322	81	25.2	72.2 (15.8)	mrs >0 35.2%	354	66	18.6	65 (13.9)	mrs >0 24%
Smith 2005[S44]	Texas, United States	381	Population-based Brain Attack Surveillance	IS	220	28	12.7			161	21	13		
Spaander 2017[S45]	Europe	9,495	Population-based registry	IS&thrombolysis	4,155	1,354	32.6	71.9 (14.5)	11 (6–17)	5,208	1,250	24	67 (13.2)	9 (5–16)
Tafreshi 2010[S46]	San Diego, United States	294	Observational clinical cohort study	IS&thrombolysis	148	51	34.5	72		146	27	18.5	68	
Tomita 2015[S47]	Japan	355	Observational clinical cohort study	Cardioembolic stroke	157	129	82	80 (8)		198	155	78	75 (9)	
Turaj 2009[S48]	Cracow, Poland	1,488	Observational clinical cohort study	IS	755	216	28.6	70.9 (13.7)	7 (3–13)	733	116	15.8	66.2 (12.7)	5 (3–10)
Vodonos 2017[S49]	Israel	1,174	Observational clinical cohort study	IS	509	148	35.1	73.8 (12.6)		665	95	17.2	66.8 (12.7)	
Zhang 2011[S50]	Guangzhou, China	669	Observational clinical cohort study	IS	220	24	10.9	39.5 (6.7)	4 (1–9)	449	20	4.5	39.2 (6.9)	5 (2–8)
Asdaghi 2016[S51]	Florida, Puerto, United States	51,317	Population-based registry	IS	25,381	4,721	18.6	73 (14.7)		25,936	4,072	15.7	68.8 (13.5)	
Buijs 2016[S52]	Groningen, the Netherlands	887	Hospital registry data	IS&thrombolysis	397	104	26	72 (13.7)	11 (7–16)	490	75	15	67.4 (11.8)	10 (6–16)
Caso 2010[S53]	Italy	1,136	Observational clinical cohort study	IS	494	110	22	76 (12.9)	9.4 (6.9)	642	108	17	70 (13)	7.6 (6.3)
de Ridder 2017[S54]	The Netherlands	5,515	Population-based registry	IS	2,778	144	19	74 (13)	6 (3–13)	2,737	153	17	70 (12)	5 (3–10)
Denti 2013[S55]	Parma, Italy	1,993	Observational clinical cohort study	IS	987	276	28	76.9 (12.7)	Scandinavian Stroke Scale 39 (2–56)	1,006	177	17.5	71.4 (13.3)	Scandinavian Stroke Scale 48 (4–58)
Dhamoon 2017[S56]	Ontario, Canada	2,5495	Population-based health care database	IS&diabetes	11,902	2,501	21	75.6 (11.5)		13,593	2,113	15.5	71.5 (11.3)	
Kim 2013[S57]	Seoul, Republic of Korea	424	Observational clinical cohort study	IS&AF	176		41.5			248		58.5		OR 2.19 (1.18–4.05)
Chiti 2015[S58]	Pisa, Italy	164	Observational clinical cohort study	IS&AF	99		60.4			65		39.6		
Benbir 2007[S59]	Instanbul, Turkey	106	Observational clinical cohort study	IS&AF	65		61.3			41		38.7		

(*continued*)

Supplementary Table S2. (Continued)

Authors and year of publication	Country and city	Total case	Study type	Population	Female					Male				
					n	No. of AF	%AF	Age	NIHSS	*n*	No. of AF	%AF	Age (years)	NIHSS
Baturova 2016[S60]	Rochester, United States	55	Observational clinical cohort study	IS&AF	19		35			36		65		
Allende 2016[S61]	Salamanca, Spain	94	Observational clinical cohort study	IS&AF	51		54.3	73.2 (14.5)		43		45.7	68.1 (12.8)	
Horner 2008[S62]	Austria	1,3831	Population-based registry	IS	6,670	2,074.37	31.1			7,161	1,489.488	20.8		
Etherton 2017[S63]	Boston, United States	319	Observational clinical cohort study	IS	131	28	21.4	68		188	23	12.2	62.7	
Colello 2018[S64]	South Carolina, United States	663	Hospital registry data	IS	354	96	27.11864			309	52	16.82848		
Niewada 2005[S65]	International	1,7370	Randomized controlled trial	IS	8,003	1,673	20.9	74		9,367	1,321	14.1	69.3	
Perera 2016[S66]	International	2,144	Global registry	Cardioembolic stroke of undetermined source	969	295	30.44376			1,175	295	25.10638		
Sakamoto 2014[S67]	Japan	244	Hospital registry data	IS&AF	124		50.81967			120		49.18033		
Salihovic 2010[S68]	Tuzia, Bosnia, and Herzegovina	2,833	Hospital registry data	IS	1,484	329	22.2	67.8 (10.6)	Scandinavian Stroke Scale 31 (15)	1,349	190	14.1	65.7 (10.5)	Scandinavian Stroke Scale 34 (15)
Seo 2015[S69]	Seoul, Republic of Korea	1,355	Hospital registry data	IS	521	162	31.09405			834	151	18.10552		
Waldenhjort 2016[S70]	Stockholm, Sweden	174	Observational clinical cohort study	Cardioembolic stroke/TIA	70	9	12.85714			104	6	5.769231		
Yesilot Barlas 2013[S71]	International	575	Population-based registry	Cardioembolic stroke	245	29	11.8			330	58	17.6		
Forster 2009[S72]	Mannheim, Germany	237	Observational clinical cohort study	IS&thrombolysis	111	21	18.9	73.9 (15.1)	12.5 (5.6)	126	18	14.3	67.8 (11.9)	11.3 (5.2)
Martin 2017[S73]	Florida, United States	221	Hospital registry data	IS&AF	137		61.99095	78.5 (11.8)		84		38.00905	71.6 (11.9)	
Nezu 2016[S74]	Japan	1,2701	Population-based registry	IS&AF	5,653		44.50831	80.3 (9.4)	14 (5–22)	7,048		55.49169	73.7 (9.9)	8 (3–18)
Lang 2017[S75]	Austria	6,3563	Population-based registry	IS	29,799	10,581	55.8			33,764	8,381	44.2		
Li 2015[S76]	Tianjing, China	810	Observational clinical cohort study	IS& aged >75 years	383	70	18.3			488	75	15.4		
Min 2017[S77]	Seoul, Republic of Korea	1,053	Observational clinical cohort study	IS	477	40	8.4	70.1 (10.7)	4.6 (2.8)	576	41	7.1	65.7 (11.9)	4.5 (2.7)
Jonsson 2015[S78]	Lund, Sweden	155	Observational clinical cohort study	IS&AF	94		61	84.8 (8.1)		61		39	79.5 (8.7)	
Cuadrado-Godia 2009[S79]	Barcelona, Spain	591	Hospital registry data	IS	273	109	39.9			318	62	19.5		
Zou 2017[S80]	Heilongjiang, China	1,070	Observational clinical cohort study	IS& aged >75 years	406	36	8.9			536	44	8.2		
Ong 2017[S81]	Taiwan	4,278	Observational clinical cohort study	IS	1,757	393	22.4	72.2 (12.2)	≥16 20.9%	2,521	373	14.8	68.3 (11.8)	≥16 13.1%
Hong 2017[S82]	Tianjing, China	951	Population-based registry	IS&AF	433		45.53102	72.6 (8.7)	13 (15)	518		54.46898	70.2 (11.1)	9 (14)
Yao 2012[S83]	Shanghai, China	1,027	Observational clinical cohort study	IS	406	30.856	7.6	71.1		621	24.219	3.9	65	
Boehme 2013[S84]	LA, TX, United States	4,925	Hospital registry data	IS	2,372	430	18.2	68 (15–100)	8 (0–40)	2,605	345	13.3	63 (26–100)	6 (0–40)
Putaala 2009[S85]	Helsinki, Finland	1,008	Observational clinical cohort study	IS	380	12	3.2			628	30	4.8		
Park 2014[S86]	Korea	9,417	Population-based registry	IS	3,958	888	22.4	71.2 (11.9)	6.7 (6.6)	5,459	867	15.9	64.8 (12.5)	5.9 (6.2)

(*continued*)

Supplementary Table S2. (Continued)

Authors and year of publication	Country and city	Total case	Study type	Population	Female					Male				
					n	No. of AF	%AF	Age	NIHSS	*n*	No. of AF	%AF	Age (years)	NIHSS
Andersen 2010[S87]	Denmark	4,0102	Population-based registry	IS	19,207	3369	17.5			20,895	2,742	13.1		
Lin 2015[S76]	Taiwan	360	Observational clinical cohort study	IS&AF	140		38.88889			220		61.11111		
Oh 2009[S88]	Seoul, Republic of Korea	18,364	Population-based registry	IS	8,092	1,319	16.3	68.4 (11.8)	6.0 (6.3)	10,542	1,386	13.1	63.4 (11.9)	5.4 (5.8)
Novello 1993[S89]	Genova, Switzerland	516	Observational clinical cohort study	IS	266	59	22.2	80.5 (11)		250	34	13.6	75.7 (9.3)	
Simpson 2005[S90]	Scotland, United Kingdom	1,120	Population-based registry	IS&AF	567		50.625			553		49.375		
Reeves 2009[S91]	United States	383,318	Population-based registry	IS	201,706	40,807	20.4	73.3		181,612	28,167	15.6	68.5	
Sjolander 2012[S92]	Sweden	18,175	Population-based registry	IS	8,923	2,553	28.6	77.8		9,252	2,373	25.6	73.4	
Yu 2015[S93]	Tianjing, China	1,484	Observational clinical cohort study	IS&aged >75 years	636	120	18.9		7.75 (0–37)	848	137	16.2		6.35 (0–36)
Ghandehari 2008[S94]	Canada and Iran	200	Observational clinical cohort study	IS	108	20	18.5			92	5	5.45		
Morin-Martin 2003[S95]	Spain	10,892	Population-based registry	IS	4,248	909	21.4	71		6,644	751	11.3	69	
Tiseo 2017[S96]	L'Aquila, Italy	285	Population-based registry	IS&AF	182		63.85965			103		36.14035		
Towfighi 2013[S97]	United States	605,960	Nationwide inpatient sample	IS&thrombolysis	332,225	73,089.5	22	76.5		273,735	48,724.83	17.8	71.2	
Gattringer 2014[S98]	Austria	47,209	Population-based registry	IS	22,329	7,190	32.3	77.9	4 (2–11)	24,880	5,623	22.7	70.3	3 (1–7)
Nathanson 2014[S99]	Stockholm, Sweden	355	Observational clinical cohort study	IS&thrombolysis	162	44	27.2	76 (67–84)		193	29	15	69 (61–76)	
Bae 2018[S100]	Korea	1,350	Population-based registry	IS&AF	621		46			729		54		
Barreira 2018[S101]	United States	1,364	Observational clinical cohort study	IS	636	246.768	38.8	67.4 (15.4)		728	227.864	31.3	62.5 (13.8)	
Burzhunova 2018[S102]	Russian	140	Observational clinical cohort study	IS&AF	71		51	74 (66–79)		69		49	67 (63–76)	
Chou 2018[S103]	Taiwan	907	Observational clinical cohort study	IS&AF	422		46.5			485		53.5		
Christensen and Christensen 2018[S104]	Denmark	1,178	Observational clinical cohort study	IS	892	188	21.1	75		286	43	15.1	69	
Hung 2018[S105]	Taiwan	403	Observational clinical cohort study	IS	170	51	21.9			233	45	26.5		
Keller 2018[S106]	Germany	292,396	Population-based registry	IS	141,801	48,495.94	34.2			150,595	39,907.68	26.5		
Radivojevic 2018[S107]	Serbia	226	Observational clinical cohort study	IS	87	34.8	40	67.5		139	31.97	23	59.5	
McGrath 2013[S108]	Canada	10,528	Population-based registry	IS	5,063	1,542	30.5			5,465	1,212	22.17749		
Antonenko 2017[S109]	Asia and Europe	1,029	Observational clinical cohort study	IS&AF	561			75.1 (9)	8.1 (7.5)	468			77.2 (9.7)	9.2 (6.9)

[a]Only 3,530 people with ischemic stroke were included in the analyses after excluding 64 cases without ECG evaluation out of a total of 3,594 cases.

Supplementary Table S3. Stroke Severity by Sex in the Included Studies

Authors and year of publication		Women	Men
Kim 2015	Moderate to severe neurological deficit	38.2%	37.5%
Martin 2017	CHADS2, mean (SD)	2.54 (1.12)	2.34 (1.41)
	CHA2DS2-VASC, mean (SD)	4.71 (1.44)	3.45 (1.83)
	mRS4–6	72.3%	46%
Nezu 2016	CHADS2, median (IQR)	2 (1–3)	2 (1–3)
	CHA2DS2-VASC, median (IQR)	4 (3–5)	3 (2–4)
	NIHSS, median (IQR)	14 (5–22)	8 (3–18)
Lang 2017	NIHSS, median (IQR)	9 (4–17)	6 (3–13)
	prestroke mRS0–1	62.4%	76.7%
Hong 2017	NIHSS, mean (SD)	13 (15)	9 (14)
Antonenko 2017	NIHSS, mean (SD)	8.1 (7.5)	9.2 (6.9)

IQR, interquartile range; NIHSS, National Institutes of Health Stroke Scale; SD, standard deviation.

Supplementary Table S4. Proportion of Antiplatelet Therapy by Sex in the Included Studies

Authors and year of publication	Women	Men
Tentschert 2004	112 (60%)	76 (40%)
Nakamura 2016	154 (51.7%)	247 (60.8%)
Martin 2017	16 (10.2%)	16 (19.1%)
Nezu 2016	1,356 (24%)	1,980 (28.1%)
Hong 2017	333 (76.9%)	396 (76.4%)
Li 2015	38 (27%)	88 (40%)
Simpson 2005	265 (46.7%)	321 (58.1%)
Reeves 2009	21,927 (88%)	17,159 (89.7%)
Sjolander 2012	352 (13.9%)	458 (19.6%)
Eriksson 2008	434 (48%)	646 (50.6%)
Antonenko 2017	141 (25.5%)	148 (31.9%)

Supplementary Table S5. Comorbidities by Sex in the Included Studies

	Authors and year of publication	Women	Men
Hypertension	Martin 2017	124 (90.5%)	65 (77.4%)
	Nezu 2016	3,803 (67.3%)	4,528 (64.3%)
	Lang 2017	9,166 (86.8%)	7,132 (85.1%)
	Hong 2017	299 (69.1%)	317 (61.2%)
	Antonenko 2017	457 (82.3%)	364 (78.3%)
Hyperlipidemia	Martin 2017	55 (40.2%)	33 (39.3%)
	Nezu 2016	1,183 (20.9%)	1,289 (18.3%)
	Lang 2017	4,956 (46.8%)	4,420 (52.7%)
	Hong 2017	129 (29.8%)	107 (20.7%)
	Antonenko 2017	161 (29.2%)	171 (36.9%)
Diabetes	Martin 2017	39 (28.5%)	34 (40.5%)
	Nezu 2016	989 (17.5%)	1,559 (22.1%)
	Lang 2017	2,759 (26.1%)	2,356 (28.1%)
	Hong 2017	114 (26.3%)	124 (23.9%)
	Antonenko 2017	144 (25.9%)	120 (25.8%)
History of stroke/transient ischemic attack	Martin 2017	36 (26.5%)	22 (26.2%)
	Nezu 2016	1,683 (29.8%)	2,237 (31.7%)
	Lang 2017	2,930 (27.7%)	2,386 (28.5%)
	Antonenko 2017	138 (25%)	127 (27.4%)
Congestive heart failure	Martin 2017	17 (12.4%)	16 (19.1%)
	Nezu 2016	375 (6.6%)	326 (4.6%)
	Antonenko 2017	99 (17.7%)	94 (20.2%)
History of myocardial infarction	Lang 2017	1,061 (10%)	1,265 (15.1%)
	Antonenko 2017	72 (13%)	94 (20.2%)

Supplementary Table S6. Comorbidities by Sex in the Included Studies

Authors and year of publication	Outcome	OR (95% CI)/proportion
Tentschert 2004	Vascular events	W vs. M 1.20 (0.80–1.90)
Kongbunkiat 2015	Hospital mortality	W vs. M 1.28 (1.18–1.39)
Giralt-Steinhauer 2012	mRS3–6 at day 90	W vs. M (57% vs. 46%)
Jonsson 2015	Hospital mortality	W vs. M (27% vs. 17%)
	1-year mortality	W vs. M (44% vs. 40%)
	5-year mortality	W vs. M (76% vs. 73%)
Hong 2017	3-month dependency	W vs. M 1.09 (0.80–1.48)
	12-month dependency	W vs. M 1.12 (0.82–1.51)
	36-month dependency	W vs. M 1.64 (1.02–2.64)
	36-month recurrence	W vs. M 2.03 (1.28–3.20)
Antonenko 2017	Mortality at 90 days	W vs. M (11.6% vs. 10.1%)
	mRS3–6 at day 90	W vs. M (57.7% vs. 41.1%)

Supplementary Table S7. Presence of Atrial Fibrillation Among People with Ischemic Stroke

Study	Study year	Total		Men	Women
		Baseline *N*	Mean age, years	AF, *n*/*N* (%)	AF, *n*/*N* (%)
Joinville, Brazil[S110]	09–14	1,555	64.5	36/811 (4.4)	46/744 (6.2)
Melbourne, Australia[S111]	96–99	915	74.7	99/431 (23.0)	131/484 (27.1)
Arcadia, Greece[S112]	93–95	375	75.1	73/212 (34.4)	69/163 (42.3)
Perth, Australia[S113]	99–00	135	76.3	15/65 (23.1)	17/70 (24.3)
Orebro, Sweden[S114]	98–00	274	75.3	26/125 (20.8)	39/149 (26.2)
Porto, Portugal[S115]	02–03	524	71.9	30/219 (13.7)	60/305 (19.7)
Auckland, New Zealand[S116]	09–14	1,015	71.8	103/499 (20.6)	128/516 (24.8)
L'Aquila, Italy[S8]	94–98	3,530[a]	78.8	340/1,676 (20.3)	529/1,854 (28.5)
Tartu, Estonia[S117]	93–95	322	72.2	40/137 (29.2)	81/195 (41.5)
Total		8,645	72.6	762/4,175 (18.3)	1,100/4,480 (24.6)

[a]Only 3,530 people with ischemic stroke were included in the analyses after excluding 64 cases without ECG evaluation out of a total of 3,594 cases.

Supplementary Table S8. Characteristics and Long-Term Outcomes of People with Ischemic Stroke and Atrial Fibrillation by Sex in the Joinville, Melbourne, Arcadia, Perth and Orebro Studies

	Joinville (*N* = 82)		Melbourne (*N* = 230)		Arcadia (*N* = 142)		Perth (*N* = 32)		Orebro (*N* = 65)	
**Study**	**Men, *n* (%)**	**Women, *n* (%)**	**Men, *n* (%)**	**Women, *n* (%)**	**Men, *n* (%)**	**Women, *n* (%)**	**Men, *n* (%)**	**Women, *n* (%)**	**Men, *n* (%)**	**Women, *n* (%)**
Sociodemographic										
Mean (SD) age	**69.4 (7.8)**	**77.8 (9.6)**	**77.8 (10.0)**	**82.3 (8.0)**	78.4 (9.5)	78.0 (8.9)	79.5 (9.7)	81.8 (9.3)	**77.2 (9.1)**	**82.3 (6.4)**
Marital status										
Single/widowed	—	—	—	—	—	—	8 (53.3)	14 (82.4)	**9 (34.6)**	**27 (69.2)**
Married							6 (40.0)	3 (17.7)	**17 (65.4)**	**11 (28.2)**
Unknown							1 (6.7)	0 (0)	**0 (0)**	**1 (2.6)**
Education level										
≤Grade 12	34 (91.4)	46 (100.0)	**46 946.5)**	**65 (49.6)**	—	—	—	—	—	—
>Grade 12	2 (5.6)	0 (0)	**49 (46.5)**	**64 (48.9)**						
Unknown	0 (0)	0 (0)	**4 (1)**	**2 (1.5)**						
Social class										
Professional	—	—	**39 (39.4)**	**36 (27.5)**	—	—	**3 (20.0)**	**0 (0)**	—	—
Nonmanual			**7 (7.1)**	**16 (12.2)**			**0 (0)**	**4 (23.5)**		
Manual			**48 (48.5)**	**46 (35.1)**			**5 (33.3)**	**3 (17.7)**		
Unknown			**5 (5.1)**	**33 (25.2)**			**7 (46.7)**	**10 (58.8)**		
Prestroke health										
In an institution										
Yes	—	—	13 (13.1)	22 (16.8)	—	—	2 (13.3)	1 (5.9)	1 (3.9)	5 (12.8)
No			85 (85.9)	108 (82.4)			13 (86.7)	15 (88.2)	25 (96.2)	34 (87.2)
Unknown			1 (1.0)	1 (0.8)			0 (0)	1 (5.9)	0 (0)	0 (0)
Modified Rankin score										
0–2	—	—	—	—	73 (100.0)	68 (95.6)	11 (73.3)	8 (47.1)	—	—
3–5					0 (0)	1 (1.5)	3 (20.0)	6 (35.3)		
Unknown					0 (0)	0 (0)	1 (6.7)	3 (17.7)		
BI score										
20	—	—	20 (20.2)	29 (22.1)	—	—	11 (73.3)	8 (47.1)	17 (65.4)	19 (48.7)
<20			46 (46.5)	56 (42.8)			3 (20.0)	6 (35.3)	0 (0)	1 (2.6)
Unknown			33 (32.3)	46 (35.1)			1 (6.7)	3 (17.7)	9 (34.6)	19 (48.7)
Mean (SD) modified Rankin score	—	—	—	—	0.4 (0.6)	0.6 (0.8)	1.4 (1.5)	1.5 (1.5)	—	—
Mean (SD) BI	—	—	18.2 (4.2)	18.4 (3.6)	—	—	18.5 (3.0)	19.1 (1.3)	20.0 (0)	19.9 (0.22)
Medical history										
Hypertension										
Yes	30 (83.3)	35 (76.1)	61 (61.6)	82 (62.6)	58 (79.5)	59 (85.5)	7 (46.7)	10 (58.8)	7 (26.9)	16 (41.0)
No	6 (16.7)	11 (23.9)	37 (37.4)	47 (35.9)	15 (20.6)	10 (14.5)	7 (46.7)	7 (41.2)	17 (65.4)	22 (56.4)
Unknown	0 (0)	0 (0)	1 (1.0)	2 (1.5)	0 (0)	0 (0)	1 (6.7)	0 (0)	2 (7.7)	1 (2.6)
Ischemic heart disease										
Yes	6 (16.7)	4 (8.7)	26 (26.3)	23 (17.6)	15 (20.6)	15 (21.7)	5 (33.3)	3 (17.7)	2 (7.7)	7 (18.0)
No	30 (83.3)	42 (91.3)	73 (73.7)	107 (81.7)	58 (79.5)	54 (78.3)	10 (66.7)	14 (82.4)	24 (92.3)	32 (82.1)
Unknown	0 (0)	0 (0)	0 (0)	1 (0.8)	0 (0)	0 (0)	0 (0)	0 (0)	0 (0)	0 (0)
Peripheral vascular disease										
Yes	—	—	**24 (24.2)**	**8 (6.1)**	3 (4.1)	5 (7.3)	—	—	9 (34.6)	18 (46.2)
No			**74 (74.8)**	**121 (92.4)**	70 (95.9)	64 (92.8)			17 (65.4)	21 (53.9)
Unknown			**1 (1.0)**	**2 (1.5)**	0 (0)	0 (0)			0 (0)	1 (0.7)

(*continued*)

Supplementary Table S8. (Continued)

Study	Joinville (*N* = 82)		Melbourne (*N* = 230)		Arcadia (*N* = 142)		Perth (*N* = 32)		Orebro (*N* = 65)	
	Men, *n* (%)	Women, *n* (%)	Men, *n* (%)	Women, *n* (%)	Men, *n* (%)	Women, *n* (%)	Men, *n* (%)	Women, *n* (%)	Men, *n* (%)	Women, *n* (%)
Transient ischemic attack										
Yes	0 (0)	1 (2.2)	10 (10.1)	12 (9.2)	20 (27.4)	13 (18.8)	**6 (40.0)**	**0 (0)**	7 (26.9)	5 (12.8)
No	36 (100)	45 (97.8)	87 (87.9)	119 (90.8)	53 (72.6)	56 (81.2)	**7 (46.7)**	**15 (88.2)**	19 (73.1)	34 (87.2)
Unknown	0 (0)	0 (0)	2 (2.0)	0 (0)	0 (0)	0 (0)	**2 (13.3)**	**2 (11.8)**	0 (0)	0 (0)
Diabetes										
Yes	—	—	18 (18.2)	20 (15.3)	21 (28.8)	24 (34.8)	5 (33.3)	2 (11.8)	5 (19.2)	4 (10.3)
No			81 (81.8)	111 (84.7)	52 (71.2)	45 (65.2)	10 (66.7)	15 (88.2)	20 (76.9)	34 (87.2)
Unknown			0 (0.0)	0 (0)	0 (0)	0 (0)	0 (0)	0 (0)	1 (3.9)	1 (2.6)
Dementia										
Yes	—	—	9 (9.1)	17 (13.0)	—	—	—	—	1 (3.9)	6 (15.4)
No			68 (68.7)	83 (63.4)					25 (96.2)	33 (84.6)
Unknown			22 (22.2)	31 (23.7)					0 (0)	0 (0.0)
Smoking										
Never	**7 (19.4)**	**36 (78.3)**	**29 (29.3)**	**70 (53.4)**	**53 (72.6)**	**68 (98.6)**	**2 (13.3)**	**11 (64.7)**	18 (69.2)	33 (84.6)
Current	**24 (66.7)**	**9 (19.6)**	**54 (54.6)**	**35 (26.7)**	**20 (27.4)**	**1 (1.5)**	**8 (53.3)**	**1 (5.9)**	5 (19.2)	6 (15.4)
Former	**5 (13.9)**	**1 (2.2)**	**8 (8.1)**	**6 (4.6)**	—	—	**0 (0)**	**1 (5.9)**	—	—
Unknown	**0 (0)**	**0 (0)**	**8 (8.1)**	**20 (15.3)**	—	—	**5 (33.3)**	**4 (23.5)**	3 (11.5)	0 (0)
Alcohol use										
Nondrinkers	**23 (63.9)**	**41 (89.1)**	**10 (10.1)**	**57 (43.5)**	**58 (79.5)**	**67 (97.1)**	4 (26.7)	8 (47.1)	—	—
Not heavy drinkers	**9 (25.0)**	**5 (10.9)**	**59 (59.6)**	**48 (36.6)**	—	—	0 (0)	3 (17.7)		
Heavy drinkers	**4 (11.1)**	**0 (0)**	**3 (3.0)**	**1 (0.8)**	**15 (20.6)**	**2 (2.9)**	3 (20.0)	1 (5.9)		
Ex-drinkers	—	—	**9 (9.1)**	**1 (0.8)**	—	—	0 (0)	0 (0)		
Unknown	**0 (0)**	**0 (0)**	**18 (18.2)**	**24 (18.3)**	—	—	8 (53.3)	5 (29.4)		
Stroke-related factors										
Hospital admission										
Yes	36 (100)	46 (100)	97 (98.0)	129 (98.5)	72 (98.6)	67 (97.1)	12 (80.0)	11 (64.7)	25 (96.2)	38 (97.4)
No	0 (0)	0 (0)	2 (2.0)	2 (1.5)	1 (1.4)	2 (2.9)	3 (20.0)	6 (35.3)	1 (3.9)	1 (2.6)
Time to arrive at hospital										
≤4.5 hours	13 (36.1)	29 (63.0)	22 (22.2)	30 (22.9)	—	—	2 (13.3)	3 (17.7)	—	—
>4.5–24 hours	14 (38.9)	8 (17.4)	4 (4.0)	12 (9.2)			0 (0)	1 (5.9)		
>24 hours	7 (19.4)	5 (10.9)	5 (5.1)	4 (3.1)			0 (0)	3 (17.7)		
Unknown	2 (5.6)	4 (8.7)	68 (68.7)	85 (64.9)			13 (96.7)	10 (58.8)		
Stroke severity										
Mean (SD) NIHSS	**7.1 (7.7)**	**11.8 (8.2)**	10.0 (8.6)	10.1 (8.5)	—	—	9.4 (7.7)	6.9 (7.4)	7.2 (6.7)	11.4 (9.6)
Median (IQR) NIHSS	**4.0 (2.0–9.0)**	**11.0 (4.0–18.0)**	8.0 (3.0–13.0)	8.0 (3.0–16.0)			6.5 (4.5–13.5)	4.0 (0–13.0)	6.0 (3.0–9.0)	8.0 (3.0–19.0)
Mean (SD) GCS, reversed	—	—	—	—	4.6 (2.8)	5.0 (3.3)	—	—	—	—
Mean (IQR) GCS, reversed					3.0 (3.0–4.0)	3.0 (3.0–4.0)				
Loss of consciousness										
Yes	—	—	24 (24.2)	34 (26.0)	5 (6.9)	10 (14.5)	—	—	—	—
No			53 (53.5)	65 (49.6)	67 (91.8)	57 (82.6)				
Unknown			22 (22.2)	32 (24.4)	1 (1.4)	2 (2.9)				

(*continued*)

Supplementary Table S8. (Continued)

Study	Joinville (*N*=82)		Melbourne (*N*=230)		Arcadia (*N*=142)		Perth (*N*=32)		Orebro (*N*=65)	
	Men, *n* (%)	Women, *n* (%)	Men, *n* (%)	Women, *n* (%)	Men, *n* (%)	Women, *n* (%)	Men, *n* (%)	Women, *n* (%)	Men, *n* (%)	Women, *n* (%)
Medications at discharge										
Antiplatelet agents										
Yes	—	—	**21 (21.2)**	**45 (34.4)**	—	—	—	—	—	—
No			**78 (78.8)**	**86 (65.7)**						
Unknown			**0 (0)**	**0 (0)**						
Anticoagulant agent										
Yes	—	—	30 (30.3)	36 (27.5)	—	—	6 (40.0)	3 (17.7)	—	—
No			69 (69.7)	95 (72.5)			6 (40.0)	8 (47.1)		
Unknown			0 (0)	0 (0)			3 (20.0)	6 (35.3)		
Outcome										
1-year mortality										
Yes	**6 (16.7)**	**20 943.5)**	42 (42.4)	55 (42.0)	32 (43.8)	28 (40.6)	5 (33.3)	6 (35.3)	9 (34.6)	19 (48.7)
No	**30 (83.3)**	**26 (56.5)**	57 (57.6)	76 (58.0)	40 (54.8)	39 (56.5)	10 (66.7)	11 (64.7)	17 (65.4)	20 (51.3)
Missing	**0 (0)**	**0 (0)**	0 (0)	0 (0)	1 (1.4)	2 (2.9)	0 (0)	0 (0)	0 (0)	0 (0)
5-year mortality										
Yes	—	—	63 (63.6)	92 (70.2)	—	—	—	—	13 (50.0)	28 (71.8)
No			36 (36.4)	39 (29.8)					13 (50.0)	11 (28.2)
Missing			0 (0)	0 (0)					0 (0)	0 (0)
Poor 1-year functional outcome[a]										
Yes (mRS >2/BI <20)	**3 (10.0)**	**8 (30.8)**	19 (33.3)	28 (36.4)	13 (32.5)	17 (43.6)	—	—	6 (35.3)	10 (50.0)
No (mRS ≤2/BI=20)	**26 (86.7)**	**14 (53.9)**	19 (33.3)	13 (16.9)	26 (65.0)	20 (51.3)			11 (64.7)	10 (50.0)
Unknown	**1 (3.3)**	**4 (15.4)**	19 (33.3)	36 (46.8)	1 (2.5)	2 (5.1)			0 (0)	0 (0)
Poor 5-year functional outcome[a]										
Yes (mRS >2/BI <20)	—	—	15 (41.7)	19 (48.7)	—	—	—	—	—	—
No (mRS ≤2/BI=20)			14 (38.9)	14 (35.9)						
Unknown			7 (19.4)	6 (15.4)						

Bold denotes statistically significant results.
[a]Among survivors only.
GCS, Glasgow Coma Scale.

Supplementary Table S9. Characteristics and Long-Term Outcomes of People with Ischemic Stroke and Atrial Fibrillation by Sex in the Porto, Auckland, L'Aquila, and Tartu Studies

	Porto (*N* = 90)		Auckland (*N* = 231)		L'Aquila (*N* = 869)		Tartu (*N* = 121)	
Study	Men, *n* (%)	Women, *n* (%)	Men, *n* (%)	Women, *n* (%)	Men, *n* (%)	Women, *n* (%)	Men, *n* (%)	Women, *n* (%)
Sociodemographic								
Mean (SD) Age	**74.6 (11.0)**	**78.6 (7.7)**	75.5 (9.8)	77.7 (12.4)	**77.1 (9.1)**	**79.9 (8.5)**	**73.5 (7.7)**	**77.1 (8.3)**
Marital status								
Single/widowed	—	—	37 (35.9)	80 (62.5)	—	—	—	—
Married			65 (63.1)	46 (35.9)				
Unknown			1 (1.0)	2 (1.6)				
Education level								
≤Grade 12	**24 (80.0)**	**33 (55.0)**	40 (38.8)	69 (53.9)	—	—	—	—
>Grade 12	**1 (3.3)**	**0 (0)**	34 (33.0)	34 (26.6)				
Unknown	**5 (16.7)**	**27 (45.0)**	29 (28.2)	25 (19.5)				
Social class								
Professional	3 (10.0)	1 (1.7)	**16 (15.5)**	**13 (10.2)**	—	—	—	—
Nonmanual	27 (90.0)	53 (88.3)	**21 (20.4)**	**33 (25.8)**				
Manual	0 (0)	3 (5.0)	**41 (39.8)**	**26 (20.3)**				
Unknown	0 (0)	3 (5.0)	**25 (24.3)**	**56 (43.8)**				
Prestroke health								
In an institution								
Yes	—	—	—	—	—	—	—	—
No								
Unknown								
Modified Rankin score								
0–2	27 (90.0)	45 (75.0)	—	—	—	—	28 (70.0)	63 (77.8)
3–5	3 (10.0)	9 (15.0)					3 (7.5)	7 (8.6)
Unknown	0 (0)	6 (10.0)					9 (22.5)	11 (13.6)
BI score								
20	—	—	—	—	—	—	—	—
<20								
Unknown								
Mean (SD) modified Rankin score	**0.6 (1.0)**	**1.2 (1.4)**	—	—	—	—	1.2 (1.0)	1.3 (0.9)
Mean (SD) BI	—	—	—	—	—	—	—	—
Medical history								
Hypertension								
Yes	17 (56.7)	40 (66.7)	66 (64.1)	79 (61.7)	**194 (57.1)**	**341 (64.5)**	**17 (42.5)**	**50 (61.7)**
No	13 (43.3)	20 (33.3)	33 (32.0)	40 (31.3)	**144 (42.4)**	**183 (34.6)**	**23 (57.7)**	**31 (38.3)**
Unknown	0 (0)	0 (0)	4 (3.9)	9 (7.0)	**2 (0.6)**	**5 (0.9)**	**0 (0)**	**0 (0)**
Ischemic heart disease								
Yes	4 (13.3)	10 (16.7)	43 (41.8)	41 (32.0)	122 (35.9)	175 (33.1)	25 (62.50	46 (56.8)
No	26 (86.7)	50 (83.3)	59 (57.3)	87 (68.0)	214 (62.9)	342 (64.7)	15 (37.5)	35 (43.2)
Unknown	0 (0)	0 (0)	1 (1.0)	0 (0)	4 (1.2)	12 (2.3)	0 (0)	0 (0)
Peripheral vascular disease								
Yes	**1 (3.3)**	**1 (1.7)**	—	—	55 (16.2)	91 (17.2)	—	—
No	**28 (93.3)**	**42 (70.0)**			282 (82.9)	422 (79.8)		
Unknown	**1 (3.3)**	**17 (28.3)**			3 (0.9)	16 (3.0)		
Transient ischemic attack								
Yes	2 (6.7)	6 (10.0)	—	—	35 (10.3)	45 (8.5)	2 (5.0)	3 (3.7)
No	28 (93.3)	54 (90.0)			298 (87.7)	464 (87.7)	38 (95.0)	78 (96.3)
Unknown	0 (0)	0 (0)			7 (2.1)	9 (3.8)	0 (0)	0 (0)
Diabetes								
Yes	—	—	—	—	—	—	—	—
No								
Unknown								
Dementia								
Yes	—	—	3 (2.9)	1 (0.8)	—	—	—	—
No			99 (96.1)	125 (97.7)				
Unknown			1 (1.0)	2 (1.6)				

(*continued*)

Supplementary Table S9. (Continued)

Study	Porto (*N* = 90)		Auckland (*N* = 231)		L'Aquila (*N* = 869)		Tartu (*N* = 121)	
	Men, *n* (%)	Women, *n* (%)	Men, *n* (%)	Women, *n* (%)	Men, *n* (%)	Women, *n* (%)	Men, *n* (%)	Women, *n* (%)
Smoking								
Never	**19 (63.3)**	**60 (100.0)**	**31 (30.1)**	**62 (48.4)**	**159 (46.8)**	**461 (87.2)**	—	—
Current	**9 (30.0)**	**0 (0)**	**57 (55.3)**	**47 (36.7)**	**150 (44.1)**	**14 (2.7)**		
Former	**2 (6.7)**	**0 (0)**	**8 (7.8)**	**8 (6.3)**	—	—		
Unknown	**0 (0)**	**0 (0)**	**7 (6.8)**	**11 (8.6)**	**31 (9.1)**	**64 (10.2)**		
Alcohol use								
Nondrinkers	**12 (40.0)**	**31 (51.7)**	**26 (25.2)**	**64 (50.0)**	—	—	—	—
Not heavy drinkers	—	—	—	—				
Heavy drinkers	**14 (46.7)**	**10 (16.7)**	**49 (47.6)**	**37 (28.9)**				
Ex-drinkers	—	—	**13 (12.6)**	**15 (11.7)**				
Unknown	**4 (13.3)**	**19 (31.7)**	**15 (14.6)**	**12 (9.4)**				
Stroke-related factors								
Hospital admission								
Yes	29 (96.7)	60 (100.0)	102 (99.0)	127 (99.2)	327 (96.2)	508 (96.0)	37 (92.5)	78 (96.3)
No	1 (3.3)	0 (0)	1 (1.0)	1 (0.8)	21 (96.0)	21 (4.0)	3 (7.5)	3 (3.7)
Time to arrive at hospital								
≤4.5 hours	13 (43.3)	30 (50.0)	77 (74.8)	108 (84.4)	—	—	11 (27.5)	29 (35.8)
>4.5–24 hours	14 (46.7)	25 (41.7)					3 (7.5)	3 (3.7)
>24 hours	1 (3.3)	0 (0)	11 (10.7)	8 (6.3)			0 (0)	1 (1.2)
Unknown	2 (6.7)	5 (8.3)	15 (14.6)	12 (9.4)			26 (65.0)	48 (59.3)
Stroke severity								
Mean (SD) NIHSS	—	—	—	—	—	—	10.4 (7.8)	11.7 (9.7)
Median (IQR) NIHSS							8.0 (4.0–19.0)	12.0 (4.0–19.0)
Mean (SD) GCS, reversed	—	—	2.2 (2.5)	2.7 (2.6)	—	—	—	—
Mean (IQR) GCS, reversed			1.0 (1.0–2.0)	1.0 (1.0–3.0)				
Loss of consciousness								
Yes	0 (0)	3 (5.0)	32 (31.1)	45 (35.2)	118 (34.7)	180 (34.0)	—	—
No	30 (100)	57 (95.0)	69 (67.0)	77 (60.2)	215 (63.2)	339 (64.1)		
Unknown	0 (0)	0 (0)	2 (1.9)	6 (4.7)	7 (2.1)	10 (1.9)		
Medications at discharge								
Antiplatelet agents								
Yes	21 (70.0)	35 (58.3)	65 (63.1)	89 (69.5)	—	—	—	—
No	9 (30.0)	25 (41.7)	38 (36.9)	37 (28.9)				
Unknown	0 (0)	0 (0)	0 (0)	0 (0)				
Anticoagulant agent								
Yes	—	—	35 (34.0)	32 (25.0)	—	—	—	—
No			68 (66.0)	94 (73.4)				
Unknown			0 (0)	2 (1.6)				
Outcome								
1-year mortality								
Yes	10 (33.3)	37 (61.7)	31 (30.1)	47 (36.7)	1,557 (46.2)	270 (51.0)	20 (50.0)	47 (58.0)
No	20 (66.8)	23 (38.3)	72 (69.9)	81 (63.3)	183 (53.8)	259 (49.0)	20 (50.0)	34 (42.0)
Missing	0 (0)	0 (0)	0 (0)	0 (0)	0 (0)	0 (0)	0 (0)	0 (0)
5-year mortality								
Yes	20 (66.7)	42 (70.0)	42 (40.8)	64 (50.0)	240 (70.6)	378 (71.5)	—	—
No	10 (33.3)	18 (30.0)	61 (59.2)	64 (50.0)	100 (29.4)	151 (28.5)		
Missing	0 (0)	0 (0)	0 (0)	0 (0)	0 (0)	0 (0)		
Poor 1-year functional outcome[a]								
Yes (mRS >2/BI <20)	11 (55.0)	14 (60.9)	—	—	—	—	6 (30.0)	13 (38.2)
No (mRS ≤2/BI = 20)	9 (45.0)	9 (39.1)					8 (40.0)	18 (52.9)
Unknown	0 (0)	0 (0)					6 (30.0)	3 (8.8)
Poor 5-year functional outcome[a]								
Yes (mRS >2/BI <20)	7 (70.0)	14 (77.8)	3 (4.9)	10 (15.6)	—	—	—	—
No (mRS ≤2/BI = 20)	3 (30.0)	2 (11.1)	13 (21.3)	12 (18.8)				
Unknown	0 (0)	2 (11.1)	45 (43.7)	43 (65.6)				

Bold denotes statistically significant results.
[a]Among survivors only.

Supplementary Table S10. Outcomes of Nine Included Cohort Studies in Patients with Ischemic Stroke and Atrial Fibrillation, by Sex

1-year mortality (nine studies)			
Yes	312 (40.9)	529 (48.1)	**0.002**
No	449 (58.9)	569 (51.7)	
Missing	1 (0.1)	2 (0.2)	
5-year mortality (five studies)			
Yes	378 (63.2)	604 (68.1)	0.051
No	220 (36.8)	283 (31.9)	
Poor 1-year functional outcome[a] (six studies)			
Yes (mRS >2/BI <20)	58 (31.5)	90 (41.3)	**0.007**
No (mRS ≤2/BI = 20)	99 (53.8)	83 (38.1)	
Unknown	27 (14.7)	45 (20.6)	
Poor 5-year functional outcome[a] (three studies)			
Yes (mRS >2/BI <20)	25 (23.4)	43 (35.5)	0.133
No (mRS ≤2/BI = 20)	30 (28.0)	28 (23.1)	
Unknown	52 (48.6)	50 (41.3)	

Bold denotes statistical significance.
[a]Among survivors only.

Supplementary Table S11. Mortality Rate Ratio Between Women and Men with Ischemic Stroke and Atrial Fibrillation at 1 Year After Stroke in Crude Models and Models with Adjustment for Age, Severity, and Prestroke Dependency

			Unadjusted	Adjusted for age		Adjusted for severity		Adjusted for prestroke dependency	
Study	Total *N*	*N*[a]	MRR (95% CI)	MRR (95% CI)	Δ%[b]	MRR (95% CI)	Δ%[b]	MRR (95% CI)	Δ%[b]
Joinville	82	82	**3.42 (1.29–9.06)**	**3.52 (1.18–10.54)**	−2	1.32 (0.43–4.09)	77	–	
Melbourne	230	230	0.95 (0.60–1.50)	0.64 (0.39–1.07)	−770	0.81 (0.43–1.55)	−310	0.86 (0.53–1.38)	−194
Arcadia	142	139	0.90 (0.53–1.51)	0.94 (0.57–1.57)	41	0.75 (0.42–1.35)	−173	–	
Perth	32	32	1.25 (0.33–4.73)	1.02 (0.26–4.03)	91	1.70 (0.38–7.51)	−138	1.24 (0.33–4.70)	4
Orebro	65	65	1.87 (0.79–4.44)	1.47 (0.59–3.67)	38	1.46 (0.56–3.82)	40	1.53 (0.60–3.94)	32
Porto	90	90	**2.79 (1.24–6.31)**	**2.46 (1.09–5.56)**	12	**2.58 (1.14–5.85)**	8	2.32 (0.99–5.47)	18
Auckland	231	179	1.07 (0.61–1.88)	0.95 (0.53–1.69)	176	0.83 (0.43–1.57)	376	1.02 (0.57–1.83)	71
L'Aquila	869	852	**1.19 (1.03–1.38)**	1.12 (0.97–1.28)	35	**1.20 (1.04–1.38)**	−5	–	
Tartu	121	111	1.19 (0.84–1.69)	1.12 (0.78–1.61)	35	1.10 (0.82–1.47)	45	1.16 (0.82–1.64)	15
Pooled	1,862	1,780	**1.24 (1.01–1.51)**	1.12 (0.89–1.39)	47	1.14 (0.98–1.32)	39	1.14 (0.90–1.43)	39
			$I^2 = 33.1\%$ $p = 0.153$	$I^2 = 40.3\%$ $p = 0.099$		$I^2 = 9.2\%$ $p = 0.358$		$I^2 = 0.0\%$ $p = 0.470$	

Bold denotes statistically significant results.
[a]The sample size was the same among the unadjusted model, age-adjusted model, and other adjusted models.
[b]% change of coefficient of sex difference in mortality rate ratio that was calculated by the formula (unadjusted $\beta -$adjusted β)/unadjusted $\beta \times 100$.
CI, confidence interval; MRR, mortality rate ratio.

Supplementary Table S12. Subgroup Analyses of Sex Difference in Mortality at 1 Year After Stroke Among Those with Ischemic Stroke and Atrial Fibrillation by the Availability of Person-Years of Follow-up

		Unadjusted				Adjusted[a]			
Person-years	No. of studies	I^2 (%)	P^H	MRR (95% CI)	$P^{subgroup}$	I^2 (%)	P^H	MRR (95% CI)	$P^{subgroup}$
Actual	7	49.6	0.064	1.38 (0.95–2.01)	0.731	0.0	0.423	0.93 (0.69–1.26)	0.314
Estimated	2[a]	0.0	0.994	1.19 (1.04–1.36)		0.0	0.675	1.12 (0.99–1.27)	

[a]The L'Aquila and Tartu studies.

Supplementary Table S13. Mortality Rate Ratio Between Women and Men with Ischemic Stroke and Atrial Fibrillation at 5 Years After Stroke in Crude Models and Models with Adjustment for Age, Severity, and Prestroke Dependency

			Unadjusted	Adjusted for age		Adjusted for severity		Adjusted for prestroke dependency	
Study	Total *N*	*N'*[a]	MRR (95% CI)	MRR (95% CI)	Δ%[b]	MRR (95% CI)	Δ%[b]	MRR (95% CI)	Δ%[b]
Melbourne	230	230	1.20 (0.80–1.78)	0.82 (0.52–1.29)	209	0.94 (0.59–1.50)	134	1.19 (0.77–1.83)	5
Orebro	65	65	2.13 (0.95–4.78)	1.69 (0.71–4.04)	31	1.91 (0.81–4.48)	14	1.89 (0.81–4.40)	16
Porto	90	90	1.41 (0.70–2.82)	1.13 (0.54–2.39)	64	1.31 (0.65–2.63)	21	1.01 (0.52–1.99)	8
Auckland	231	179	1.13 (0.71–1.78)	1.01 (0.64–1.60)	92	0.93 (0.57–1.53)	159	1.09 (0.69–1.73)	29
L'Aquila	869	852	1.11 (1.01–1.21)	1.05 (0.97–1.14)	53	1.11 (1.02–1.21)	0	—	
Pooled	1,485	564	1.12 (1.03–1.22)	1.05 (0.96–1.13)	57	1.07 (0.81–1.43)	40	1.18 (0.90–1.54)	−46
			$I^2=0.0\%$ $p=0.556$	$I^2=0.0\%$ $p=0.670$		$I^2=0.0\%$ $p=0.446$		$I^2=0.0\%$ $p=0.678$	

Bold denotes statistically significant results.
[a]The sample size was the same among the unadjusted model, age-adjusted model, and fully adjusted model.
[b]% change of coefficient of sex difference in mortality rate ratio that was calculated by the formula (unadjusted β−adjusted β)/unadjusted $\beta \times 100$.

Supplementary Table S14. Relative Risk of Having Poorer Functional Outcome for Women Survivors with Ischemic Stroke and Atrial Fibrillation Compared with Men at 1 Year After Stroke in Crude Models and Models with Adjustment for Age, Severity, and Prestroke Dependency

			Unadjusted	Adjusted for age		Adjusted for severity		Adjusted for prestroke dependency	
Study	Total *N*	*N'*[a]	RR (95% CI)	RR (95% CI)	Δ%[b]	RR (95% CI)	Δ%[b]	RR (95% CI)	Δ%[b]
Joinville	51	51	**3.52 (1.04–11.9)**	1.43 (0.45–4.51)	72	3.09 (0.74–12.9)	10	—	
Melbourne	78	74	1.40 (0.96–2.05)	1.25 (0.86–1.82)	34	1.3 4 (0.93–1.94)	13	**1.55 (1.02–2.34)**	−30
Arcadia	76	76	1.38 (0.78–2.43)	1.40 (0.79–2.45)	−5	1.42 (0.81–2.46)	−9	—	
Orebro	37	37	1.42 (0.64–3.12)	1.04 (0.52–2.10)	89	1.32 (0.65–2.68)	21	1.49 (0.68–3.27)	−14
Porto	43	43	1.11 (0.66–1.86)	1.15 (0.69–1.92)	−34	1.11 (0.66–1.86)	0	1.06 (0.65–1.72)	44
Tartu	45	41	0.95 (0.44–2.05)	0.84 (0.41–1.72)	−240	0.84 (0.39–1.83)	−240	0.82 (0.40–1.67)	−287
Pooled	330	322	**1.33 (1.05–1.68)**	1.19 (0.94–1.49)	39	**1.28 (1.01–1.61)**	13	1.24 (0.96–1.61)	25
			$I^2=0.0\%$ $p=0.582$	$I^2=0.0\%$ $p=0.906$		$I^2=0.0\%$ $p=0.692$		$I^2=0.0\%$ $p=0.431$	

Bold denotes statistically significant results.
[a]The sample size was the same among the unadjusted model, age-adjusted model, and fully adjusted model.
[b]% change of coefficient of sex difference in mortality rate ratio that was calculated by the formula (unadjusted β−adjusted β)/unadjusted $\beta \times 100$.
RR, relative risk.

Supplementary Table S15. Relative Risk of Having Poorer Functional Outcome for Women Survivors with Ischemic Stroke and Atrial Fibrillation Compared with Men at 5 Years After Stroke in Crude Models and Models with Adjustment for Age, Severity, and Prestroke Dependency

			Unadjusted	Adjusted for age		Adjusted for severity		Adjusted for prestroke dependency	
Study	Total *N*	*N'*[a]	RR (95% CI)	RR (95% CI)	Δ%[b]	RR (95% CI)	Δ%[b]	RR (95% CI)	Δ%[b]
Melbourne	62	62	1.11 (0.70–1.77)	1.01 (0.64–1.60)	90	1.11 (0.70–1.77)	0	1.15 (0.69–1.91)	−34
Porto	26	26	1.25 (0.79–1.97)	1.27 (0.82–2.00)	−7	1.25 (0.79–1.97)	0	1.27 (0.81–2.00)	−7
Auckland	38	27	2.35 (0.60–9.20)	1.80 (0.45–7.22)	82	2.29 (0.58–9.06)	77	1.79 (0.42–7.63)	76
Pooled	126	115	1.22 (0.89–1.68)	1.17 (0.86–1.58)	21	1.22 (0.89–1.68)	0	**1.23 (0.90–1.69)**	−4
			$I^2=0.0\%$ $p=0.591$	$I^2=0.0\%$ $p=0.639$		$I^2=0.0\%$ $p=0.211$		$I^2=0.0\%$ $p=0.835$	

Bold denotes statistically significant results.
[a]The sample size was the same among the unadjusted model, age-adjusted model, and fully adjusted model.
[b]% change of coefficient of sex difference in mortality rate ratio that was calculated by the formula (unadjusted β−adjusted β)/unadjusted $\beta \times 100$.

Supplementary Table S16. Relative Risk of Having Poorer Outcome (Either Mortality or Poor Functional Outcome [Modified Rankin Scale >2 or Barthel Index <20]) *at 1 Year After Stroke* Among Survivors with Ischemic Stroke and Atrial Fibrillation in Crude Models and Adjusted Models Among Studies with Mortality and Functional Outcome Data

		Unadjusted	Adjusted for age		Adjusted for severity		Adjusted for prestroke dependency		Fully adjusted	
Study	N[a]	RR (95% CI)	RR (95% CI)	Δ%[b]	RR (95% CI)	Δ%[b]	RR (95% CI)	Δ%[b]	RR (95% CI)	Δ%[b]
Joinville	77	**2.59 (1.41–4.75)**	**2.12 (1.09–4.10)**	21	**2.01 (1.17–3.47)**	27	—		1.72 (0.92–3.20)	43
Melbourne	175	1.13 (0.98–1.31)	1.07 (0.93–1.22)	45	1.11 (0.97–1.28)	15	1.15 (0.99–1.32)	−14	1.07 (0.95–1.23)	45
Arcadia	136	1.09 (0.86–1.39)	1.10 (0.87–1.39)	−11	1.06 (0.84–1.34)	32	—		1.06 (0.84–1.33)	32
Orebro	65	1.29 (0.88–1.89)	1.09 (0.78–1.52)	66	1.13 (0.80–1.58)	52	1.27 (0.87–1.85)	6	0.98 (0.72–1.33)	108
Porto	90	**1.83 (1.14–2.94)**	**1.75 (1.08–2.83)**	7	**1.73 (1.08–2.78)**	9	**1.61 (1.00–2.60)**	21	1.47 (0.91–2.36)	36
Tartu	105	1.01 (0.81–1.27)	0.95 (0.75–1.19)	615	0.98 (0.80–1.20)	303	0.99 (0.80–1.23)	201	0.93 (0.75–1.15)	829
Pooled	648	**1.25 (1.04–1.50)**	1.13 (0.97–1.32)	45	1.16 (0.99–1.34)	33	1.14 (0.99–1.32)	41	1.06 (0.95–1.18)	74
		$I^2=12.3\%$ $p=0.030$	$I^2=9.2\%$ $p=0.101$		$I^2=48.1\%$ $p=0.086$		$I^2=23.5\%$ $p=0.270$		$I^2=15.2\%$ $p=0.317$	

Bold denotes statistically significant results.

[a]The sample size was the same among the unadjusted model, age-adjusted model, and fully adjusted model.

[b]% change of coefficient of sex difference in mortality rate ratio that was calculated by the formula (unadjusted $\beta-$adjusted β)/unadjusted $\beta\times100$.

Supplementary Table S17. Relative Risk of Having Poorer Outcome (Either Died or Poorly Functioned [Modified Rankin Scale >2 or Barthel Index <20]) *at 5 Years After Stroke* Among Survivors with Ischemic Stroke and Atrial Fibrillation in Crude Models and Adjusted Models Among Studies with Mortality and Functional Outcome Data

		Unadjusted	Adjusted for age		Adjusted for severity		Adjusted for prestroke dependency		Fully adjusted	
Study	N^a	RR (95% CI)	RR (95% CI)	$\Delta\%^b$	RR (95% CI)	$\Delta\%^b$	RR (95% CI)	$\Delta\%^b$	RR (95% CI)	$\Delta\%^b$
Melbourne	217	1.05 (0.94–1.17)	0.99 (0.89–1.10)	121	1.05 (0.94–1.16)	0	1.06 (0.95–1.18)	−19	1.00 (0.91–1.12)	100
Porto	72	**1.76 (1.16–2.67)**	**1.16 (1.07–2.42)**	73	1.64 (1.09–2.48)	12	1.65 (1.07–2.55)	11	1.43 (0.94–2.17)	37
Auckland	110	1.05 (0.89–1.24)	1.01 (0.86–1.18)	80	1.04 (0.88–1.22)	20	1.02 (0.87–1.20)	59	0.99 (0.85–1.16)	121
Pooled	399	1.18 (0.94–1.49)	1.11 (0.90–1.38)	37	1.15 (0.93–1.42)	16	1.14 (0.93–1.39)	21	1.06 (0.90–1.25)	65
		$I^2=8.6\%$ $p=0.014$	$I^2=8.0\%$ $p=0.019$		$I^2=72.5\%$ $p=0.026$		$I^2=69.6\%$ $p=0.037$		$I^2=4.9\%$ $p=0.085$	

Bold denotes statistically significant results.

[a]The sample size was the same among the unadjusted model, age-adjusted model, and fully adjusted model.

[b]% change of coefficient of sex difference in mortality rate ratio that was calculated by the formula (unadjusted β−adjusted β)/unadjusted $\beta \times 100$.

Supplementary References

S1. Cabral NL, Goncalves ARR, Longo AL, et al. Incidence of stroke subtypes, prognosis and prevalence of risk factors in Joinville, Brazil: A 2 year community based study. J Neurol Neurosurg Psychiatry 2009;80:755.

S2. Sturm JW, Donnan GA, Dewey HM, Macdonell RA, Gilligan AK, Thrift AG. Determinants of handicap after stroke: The North East Melbourne Stroke Incidence Study (NEMESIS). Stroke 2004;35:715–720.

S3. Vemmos KN, Bots ML, Tsibouris PK, et al. Stroke incidence and case fatality in southern Greece: The Arcadia stroke registry. Stroke 1999;30:363–370.

S4. Anderson CS, Jamrozik KD, Broadhurst RJ, Stewart-Wynne EG. Predicting survival for 1 year among different subtypes of stroke. Results from the Perth Community Stroke Study. Stroke 1994;25:1935–1944.

S5. Appelros P, Nydevik I, Seiger Å, Terént A. High incidence rates of stroke in Örebro, Sweden: Further support for regional incidence differences within Scandinavia. Cerebrovasc Dis 2002;14:161–168.

S6. Correia M, Silva MR, Matos I, et al. Prospective community-based study of stroke in Northern Portugal: Incidence and case fatality in rural and urban populations. Stroke 2004;35:2048–2053.

S7. Feigin V, Carter K, Hackett M, et al. Ethnic disparities in incidence of stroke subtypes: Auckland Regional Community Stroke Study, 2002–2003. Lancet Neurol 2006;5:130–139.

S8. Carolei A, Marini C, Di Napoli M, et al. High stroke incidence in the prospective community-based L'Aquila registry (1994–1998). First year's results. Stroke 1997;28:2500–2506.

S9. Marini C, De Santis F, Sacco S, et al. Contribution of atrial fibrillation to incidence and outcome of ischemic stroke: Results from a population-based study. Stroke 2005;36:1115–1119.

S10. Kôrv J, Roose M, Kaasik A-E. Stroke Registry of Tartu, Estonia, from 1991 through 1993. Cerebrovasc Dis 1997;7:154–162.

S11. Kent DM, Price LL, Ringleb P, Hill MD, Selker HP. Sex-based differences in response to recombinant tissue plasminogen activator in acute ischemic stroke: A pooled analysis of randomized clinical trials. Stroke 2005;36:62–65.

S12. Lasek-Bal A, Szymszal J, Kazibutowska Z. Stroke risk factors, course and long-term functional outcome of first-ever stroke in women. Open Access Maced J Med Sci 2014;2:119–123.

S13. Akanksha G, Paramdeep K, Gagandeep S, et al. Clinical features, risk factors, and short-term outcome of ischemic stroke, in patients with atrial fibrillation: Data from a population-based study. Ann Indian Acad Neurol 2017;20:289–293.

S14. Yesilot NF, Koyuncu BA, Coban O, Tuncay R, Bahar SZ. Gender differences in acute stroke: Istanbul medical school stroke registry. Neurol India 2011;59:174–179.

S15. Irie F, Kamouchi M, Hata J, et al. Sex differences in short-term outcomes after acute ischemic stroke: The fukuoka stroke registry. Stroke 2015;46:471–476.

S16. Huang Y, Wang J, Wei JW, et al. Age and gender variations in the management of ischaemic stroke in China. Int J Stroke 2010;5:351–359.

S17. Hametner C, MacIsaac RL, Kellert L, et al. Sex and stroke in thrombolyzed patients and controls. Stroke 2017;48:367–374.

S18. Hametner C, Kellert L, Ringleb PA. Impact of sex in stroke thrombolysis: A coarsened exact matching study. BMC Neurol 2015;15:10.

S19. Gray LJ, Sprigg N, Bath PM, et al. Sex differences in quality of life in stroke survivors: Data from the Tinzaparin in Acute Ischaemic Stroke Trial (TAIST). Stroke 2007;38:2960–2964.

S20. Nakamura A, Kuroda J, Ago T, et al. Causes of ischemic stroke in patients with non-valvular atrial fibrillation. Cerebrovasc Dis 2016;42:196–204.

S21. Munoz-Rivas N, Mendez-Bailon M, Hernandez-Barrera V, et al. Time trends in ischemic stroke among type 2 diabetic and non-diabetic patients: Analysis of the Spanish national hospital discharge data (2003–2012). PLoS One 2015;10:e0145535.

S22. Martin-Merino E, Ruigomez A, Johansson S, Garcia-Rodriguez LA. Hospitalised ischaemic cerebrovascular accident and risk factors in a primary care database. Pharmacoepidemiol Drug Saf 2011;20:1050–1056.

S23. Kongbunkiat K, Kasemsap N, Travanichakul S, Thepsuthammarat K, Tiamkao S, Sawanyawisuth K. Hospital mortality from atrial fibrillation associated with ischemic stroke: A national data report. Int J Neurosci 2015;125:924–928.

S24. Giralt-Steinhauer E, Cuadrado-Godia E, Ois A, et al. CHA<inf>2</inf>DS<inf>2</inf>-VASc score and prognosis in ischemic strokes with atrial fibrillation. J Neurol 2012;259:745–751.

S25. Kim TW, Jung SW, Song IU, et al. Left atrial dilatation is associated with severe ischemic stroke in men with non-valvular atrial fibrillation. J Neurol Sci 2015;354:97–102.

S26. Heuschmann PU, Kolominsky-Rabas PL, Misselwitz B, et al. Predictors of in-hospital mortality and attributable risks of death after ischemic stroke: The German Stroke Registers Study Group. Arch Intern Med 2004;164:1761–1768.

S27. Candelise L, Pinardi G, Morabito A. Mortality in acute stroke with atrial fibrillation. The Italian Acute Stroke Study Group. Stroke 1991;22:169–174.

S28. Kapral MK, Devon J, Winter AL, Wang J, Peters A, Bondy SJ. Gender differences in stroke care decision-making. Med Care 2006;44:70–80.

S29. Kapral MK, Ben-Yakov M, Fang J, et al. Gender differences in carotid imaging and revascularization following stroke. Neurology 2009;73:1969–1974.

S30. Sen Lin S, Lin S, Wu B, et al. Characteristics, treatment and outcome of ischemic stroke with atrial fibrillation in a Chinese hospital-based stroke study. Cerebrovasc Dis 2011;31:419–426.

S31. Lisabeth LD, Brown DL, Hughes R, Majersik JJ, Morgenstern LB. Acute stroke symptoms: Comparing women and men. Stroke 2009;40:2031–2036.

S32. Lisabeth LD, Roychoudhury C, Brown DL, Levine SR. Do gender and race impact the use of antithrombotic therapy in patients with stroke/TIA? Neurology 2004;62:2313–2315.

S33. Lorenzano S, Ahmed N, Falcou A, et al. Does sex influence the response to intravenous thrombolysis in ischemic stroke?: Answers from safe implementation of treatments in Stroke-International Stroke Thrombolysis Register. Stroke 2013;44:3401–3406.
S34. Martinez-Sanchez P, Fuentes B, Fernandez-Dominguez J, et al. Young women have poorer outcomes than men after stroke. Cerebrovasc Dis 2011;31:455–463.
S35. McDermott M, Lisabeth LD, Baek J, et al. Sex disparity in stroke quality of care in a community-based study. J Stroke Cerebrovasc Dis 2017;26:1781–1786.
S36. Meseguer E, Mazighi M, Labreuche J, et al. Outcomes of intravenous recombinant tissue plasminogen activator therapy according to gender: A clinical registry study and systematic review. Stroke 2009;40:2104–2110.
S37. Mizrahi EH, Waitzman A, Arad M, Adunsky A. Atrial fibrillation predicts cognitive impairment in patients with ischemic stroke. Am J Alzheimers Dis Other Demen 2011;26:623–626.
S38. Nagaraja N, Bhattacharya P, Mada F, et al. Gender based differences in acute stroke care in Michigan hospitals. J Neurol Sci 2012;314:88–91.
S39. Mizrahi EH, Waitzman A, Arad M, Adunsky A. Gender and the functional outcome of elderly ischemic stroke patients. Arch Gerontol Geriatr 2012;55:438–441.
S40. Olsen TS, Andersen KK. Female survival advantage relates to male inferiority rather than female superiority: A hypothesis based on the impact of age and stroke severity on 1-week to 1-year case fatality in 40,155 men and women. Gend Med 2010;7:284–295.
S41. Padjen V, Bodenant M, Jovanovic DR, et al. Outcome of patients with atrial fibrillation after intravenous thrombolysis for cerebral ischaemia. J Neurol 2013;260:3049–3054.
S42. Rodriguez-Campello A, Jimenez-Conde J, Ois A, et al. Sex-related differences in abdominal obesity impact on ischemic stroke risk. Eur J Neurol 2017;24:397–403.
S43. Silva GS, Lima FO, Camargo EC, et al. Gender differences in outcomes after ischemic stroke: Role of ischemic lesion volume and intracranial large-artery occlusion. Cerebrovasc Dis 2010;30:470–475.
S44. Smith MA, Lisabeth LD, Brown DL, Morgenstern LB. Gender comparisons of diagnostic evaluation for ischemic stroke patients. Neurology 2005;65:855–858.
S45. Spaander FH, Zinkstok SM, Baharoglu IM, et al. Sex differences and functional outcome after intravenous Thrombolysis. Stroke 2017;48:699–703.
S46. Tafreshi GM, Raman R, Ernstrom K, Meyer BC, Hemmen TM. Gender differences in acute stroke treatment: The University of California San Diego experience. Stroke 2010;41:1755–1757.
S47. Tomita H, Hagii J, Metoki N, et al. Impact of sex difference on severity and functional outcome in patients with cardioembolic stroke. J Stroke Cerebrovasc Dis 2015;24:2613–2618.
S48. Turaj W, Slowik A, Wnuk M, Szczudlik A. Gender-related differences in diagnostic evaluation and outcome of ischemic stroke in Poland. Stroke 2009;40:980–982.
S49. Vodonos A, Novack V, Horev A, Abu Salameh I, Lotan Y, Ifergane G. Do gender and season modify the triggering effect of ambient temperature on ischemic stroke? Womens Health Issues 2017;27:245–251.
S50. Zhang B, Pu S, Zhang W, et al. Sex differences in risk factors, etiology, and short-term outcome of cerebral infarction in young patients. Atherosclerosis 2011;216:420–425.
S51. Asdaghi N, Romano JG, Wang K, et al. Sex Disparities in ischemic stroke care: FL-PR CReSD Study (Florida-Puerto Rico Collaboration to reduce stroke disparities). Stroke 2016;47:2618–2626.
S52. Buijs JE, Uyttenboogaart M, Brouns R, de Keyser J, Kamphuisen PW, Luijckx GJ. The effect of age and sex on clinical outcome after intravenous recombinant tissue plasminogen activator treatment in patients with acute ischemic stroke. J Stroke Cerebrovasc Dis 2016;25:312–316.
S53. Caso V, Paciaroni M, Agnelli G, et al. Gender differences in patients with acute ischemic stroke. Womens Health (Lond) 2010;6:51–57.
S54. de Ridder I, Kuramatsu J, Gerner S, et al. No sex differences in long-term functional outcome after intracerebral hemorrhage. Int J Stroke 2017;12:416–420.
S55. Denti L, Artoni A, Scoditti U, et al. Impact of gender-age interaction on the outcome of ischemic stroke in an Italian cohort of patients treated according to a standardized clinical pathway. Eur J Intern Med 2013;24:807–812.
S56. Dhamoon MS, Liang JW, Zhou L, Stamplecoski M, Kapral MK, Shah BR. Sex differences in outcomes after stroke in patients with diabetes in Ontario, Canada. J Stroke Cerebrovasc Dis 2018;27:210–220.
S57. Kim SJ, Ryoo S, Kwon S, et al. Is atrial fibrillation always a culprit of stroke in patients with atrial fibrillation plus stroke? Cerebrovasc Dis 2013;36:373–382.
S58. Chiti A, Giannini N, Terni E, et al. "Cardioembolic profile" in patients with ischemic stroke: Data from the analysis of 1037 cases. J Stroke Cerebrovasc Dis 2015;24:1287–1290.
S59. Benbir G, Uluduz D, Ince B, Bozluolcay M. Atherothrombotic ischemic stroke in patients with atrial fibrillation. Clin Neurol Neurosurg 2007;109:485–490.
S60. Baturova MA, Sheldon SH, Carlson J, et al. Electrocardiographic and Echocardiographic predictors of paroxysmal atrial fibrillation detected after ischemic stroke. BMC Cardiovasc Dis 2016;16:209.
S61. Allende M, Molina E, Gonzalez-Porras JR, Toledo E, Lecumberri R, Hermida J. Short leukocyte telomere length is associated with cardioembolic stroke risk in patients with atrial fibrillation. Stroke 2016;47:863–865.
S62. Horner S, Niederkorn K, Schnabl S, Fazekas F, MitarbeiterInnen am Osterreichischen Stroke-Unit Register und in Zusammenarbeit mit der Gesundheit Osterreich GmbH, Geschäftsbereich BIQS. [Gender aspects of ischemic stroke. An analysis of the Austrian Stroke-Unit Registry]. Wien Med Wochenschr 2008;158:446–452.
S63. Etherton MR, Wu O, Cougo P, et al. Structural integrity of normal appearing white matter and sex-specific outcomes after acute ischemic stroke. Stroke 2017;48:3387–3389.
S64. Colello MJ, Ivey LE, Gainey J, et al. Pharmacological thrombolysis for acute ischemic stroke treatment: Gender differences in clinical risk factors. Adv Med Sci 2018;63:100–106.
S65. Niewada M, Kobayashi A, Sandercock PA, Kaminski B, Czlonkowska A, International Stroke Trial Collaborative Group. Influence of gender on baseline features and clinical outcomes among 17,370 patients with confirmed ischaemic stroke in the international stroke trial. Neuroepidemiology 2005;24:123–128.
S66. Perera KS, Vanassche T, Bosch J, et al. Global survey of the frequency of atrial fibrillation-associated stroke: Embolic stroke of undetermined source global registry. Stroke 2016;47:2197–2202.
S67. Sakamoto Y, Sato S, Kuronuma Y, Nagatsuka K, Minematsu K, Toyoda K. Factors associated with proximal carotid axis occlusion in patients with acute stroke and atrial fibrillation. J Stroke Cerebrovasc Dis 2014;23:799–804.
S68. Salihovic D, Smajlovic D, Sinanovic O, Kojic B. Sex differences in patients with acute ischemic stroke in Tuzla region, Bosnia and Herzegovina. Bosn J Basic Med Sci 2010;10:116–120.
S69. Seo WK, Kang SH, Jung JM, Choi JY, Oh K. Novel composite score to predict atrial Fibrillation in acute stroke patients: AF predicting score in acute stroke. Int J Cardiol 2016;209:184–189.
S70. Waldenhjort D, Sobocinski Doliwa P, Alam M, et al. Echocardiographic measures of atrial function may predict atrial fibrillation in stroke patients. Scand Cardiovasc J 2016;50:236–242.
S71. Yesilot Barlas N, Putaala J, Waje-Andreassen U, et al. Etiology of first-ever ischaemic stroke in European young adults: The 15 cities young stroke study. Eur J Neurol 2013;20:1431–1439.
S72. Forster A, Gass A, Kern R, et al. Gender differences in acute ischemic stroke: Etiology, stroke patterns and response to thrombolysis. Stroke 2009;40:2428–2432.
S73. Martin RC, Burgin WS, Schabath MB, et al. Gender-specific differences for risk of disability and death in atrial fibrillation-related stroke. Am J Cardiol 2017;119:256–261.
S74. Nezu T, Hosomi N, Kondo K, et al. Greater severity of neurological defects in women admitted with atrial fibrillation-related stroke. Circ J 2016;80:250–255.
S75. Lang C, Seyfang L, Ferrari J, et al. Do women with atrial fibrillation experience more severe strokes?: Results from the Austrian Stroke Unit Registry. Stroke 2017;48:778–780.
S76. Li B, Wang T, Lou Y, et al. Sex differences in outcomes and associated risk factors after acute ischemic stroke in elderly patients: A prospective follow-up study. J Stroke Cerebrovasc Dis 2015;24:2277–2284.

S77. Min K, Kwon S, Cho SY, et al. Atrial fibrillation is strongly associated with the neutrophil to lymphocyte ratio in acute ischemic stroke patients: A retrospective study. J Clin Lab Anal 2017;31:e22041.
S78. Jonsson AC, Ek J, Kremer C. Outcome of men and women after atrial fibrillation and stroke. Acta Neurol Scand 2015;132:125–131.
S79. Cuadrado-Godia E, Jimenez-Conde J, Ois A, Rodriguez-Campello A, Garcia-Ramallo E, Roquer J. Sex differences in the prognostic value of the lipid profile after the first ischemic stroke. J Neurol 2009;256:989–995.
S80. Zou C, Wei C, Wang Z, Jin Y. Sex differences in outcomes and risk factors among elderly patients with ischemic stroke. Oncotarget 2017;8:104582–104593.
S81. Ong CT, Wong YS, Sung SF, et al. Sex-related differences in the risk factors for in-hospital mortality and outcomes of ischemic stroke patients in rural areas of Taiwan. PLoS One 2017;12:e0185361.
S82. Hong Y, Yang X, Zhao W, et al. Sex differences in outcomes among stroke survivors with non-valvular atrial fibrillation in China. Front Neurol 2017;8:166.
S83. Yao XY, Lin Y, Geng JL, et al. Age- and gender-specific prevalence of risk factors in patients with first-ever ischemic stroke in China. Stroke Res Treat 2012;2012:136398.
S84. Boehme AK, Siegler JE, Mullen MT, et al. Racial and gender differences in stroke severity, outcomes, and treatment in patients with acute ischemic stroke. J Stroke Cerebrovasc Dis 2014;23:e255–e261.
S85. Putaala J, Metso AJ, Metso TM, et al. Analysis of 1008 consecutive patients aged 15 to 49 with first-ever ischemic stroke: The Helsinki young stroke registry. Stroke 2009;40:1195–1203.
S86. Park TH, Ko Y, Lee SJ, et al. Gender differences in the age-stratified prevalence of risk factors in Korean ischemic stroke patients: A nationwide stroke registry-based cross-sectional study. Int J Stroke 2014;9:759–765.
S87. Andersen KK, Andersen ZJ, Olsen TS. Age- and gender-specific prevalence of cardiovascular risk factors in 40,102 patients with first-ever ischemic stroke: A Nationwide Danish Study. Stroke 2010;41:2768–2774.
S88. Oh MS, Yu KH, Roh JK, Lee BC. Gender differences in the mortality and outcome of stroke patients in Korea. Cerebrovasc Dis 2009;28: 427–434.
S89. Novello P, Ajmar G, Bianchini D, et al. Ischemic stroke and atrial fibrillation. A clinical study. Ital J Neurol Sci 1993;14:571–576.
S90. Simpson CR, Wilson C, Hannaford PC, Williams D. Evidence for age and sex differences in the secondary prevention of stroke in Scottish primary care. Stroke 2005;36:1771–1775.
S91. Reeves MJ, Fonarow GC, Zhao X, Smith EE, Schwamm LH. Quality of care in women with ischemic stroke in the GWTG program. Stroke 2009;40:1127–1133.
S92. Sjolander M, Eriksson M, Glader EL. Few sex differences in the use of drugs for secondary prevention after stroke: A nationwide observational study. Pharmacoepidemiol Drug Saf 2012;21:911–919.
S93. Yu C, An Z, Zhao W, et al. Sex differences in stroke subtypes, severity, risk factors, and outcomes among elderly patients with acute ischemic stroke. Front Aging Neurosci 2015;7:174.
S94. Ghandehari K, Shuaib A, Ardalani GF. The frequency rate of cardioembolic etiology in North American and Iranian stroke patients: A pilot double-center study. Iran Red Crescent Med J 2008;10:66–68.
S95. Morin-Martin M, Gonzalez-Santiago R, Gil-Nunez AC, Vivancos-Mora J. [Women and strokes. Hospital epidemiology in Spain]. Rev Neurol 2003;37:701–705.
S96. Tiseo C, Ornello R, Degan D, et al. Time trends of atrial fibrillation prevalence in patients with first ever ischemic stroke. Eur Stroke J 2017;2 (1 Supplement 1):191–192.
S97. Towfighi A, Markovic D, Ovbiagele B. Sex differences in revascularization interventions after acute ischemic stroke. J Stroke Cerebrovasc Dis 2013;22:e347–e353.
S98. Gattringer T, Ferrari J, Knoflach M, et al. Sex-related differences of acute stroke unit care: Results from the Austrian stroke unit registry. Stroke 2014;45:1632–1638.
S99. Nathanson D, Patrone C, Nystrom T, Von Euler M. Sex, diastolic blood pressure, and outcome after thrombolysis for ischemic stroke. Stroke Res Treat 2014;2014:747458.
S100. Bae HJ, Heo JH, Jung KH, et al. Antithrombotic treatments in patients with acute ischemic stroke and non-valvular atrial fibrillation before introduction of non-Vitamin K antagonist oral anticoagulants into practice in Korea. PLoS One 2018;13:e0202803.
S101. Barreira CM, Bouslama M, Al-Bayati AR, et al. Endovascular therapy and gender disparities in stroke outcomes. Eur Stroke J 2018;3 (1 Supplement 1):56.
S102. Burzhunova M, Alieva M, Geraskina L, Garabova N, Fonyakin A. Gender differences in patients with stroke and atrial fibrillation. Eur Stroke J 2018;3 (1 Supplement 1):404.
S103. Chou PS, Ho BL, Chan YH, Wu MH, Hu HH, Chao AC. Delayed diagnosis of atrial fibrillation after first-ever stroke increases recurrent stroke risk: A 5-year nationwide follow-up study. Int Med J 2018;48:661–667.
S104. Christensen L, Christensen H. Exploring the sex differences in atrial fibrillation and stroke in a cohort of 1178 patients with 10 year follow up. Eur Stroke J 2018;3 (1 Supplement 1):511.
S105. Hung KH, Lai JC, Hsu KN, et al. Gender gap and risk factors for poor stroke outcomes: A single hospital-based prospective cohort study. J Stroke Cerebrovasc Dis 2018;27:2250–2258.
S106. Keller K, Geyer M, Munzel T, Ostad MA. Gender-differences in prevalence and outcome of ischemic stroke and promoting factors of atrial thrombi. Artery Res 2018;22:68–78.
S107. Radivojevic A, Budimkic M, Stanarcevic P, et al. Dowomen suffer more from stroke? Eur Stroke J 2018;3 (1 Supplement 1):512–513.
S108. McGrath ER, Kapral MK, Fang J, et al. Association of atrial fibrillation with mortality and disability after ischemic stroke. Neurology 2013;81:825–832.
S109. Antonenko K, Paciaroni M, Agnelli G, et al. Sex-related differences in risk factors, type of treatment received and outcomes in patients with atrial fibrillation and acute stroke: Results from the RAF-study (early recurrence and cerebral bleeding in patients with acute ischemic stroke and atrial fibrillation). Eur Stroke J 2017;2:46–53.
S110. Moro CH, Goncalves AR, Longo AL, et al. Trends of the incidence of ischemic stroke thrombolysis over seven years and one-year outcome: A population-based study in Joinville, Brazil. Cerebrovasc Dis Extra 2013;3:156–166.
S111. Gall SL, Donnan G, Dewey HM, et al. Sex differences in presentation, severity, and management of stroke in a population-based study. Neurology 2010;74:975–981.
S112. Vemmos KN, Bots ML, Tsibouris PK, et al. Prognosis of stroke in the south of Greece: 1 year mortality, functional outcome and its determinants: The Arcadia Stroke Registry. J Neurol Neurosurg Psychiatry 2000;69:595–600.
S113. Hankey GJ, Jamrozik K, Broadhurst RJ, et al. Five-year survival after first-ever stroke and related prognostic factors in the Perth Community Stroke Study. Stroke 2000;31:2080–2086.
S114. Appelros P, Nydevik I, Seiger A, Terent A. High incidence rates of stroke in Orebro, Sweden: Further support for regional incidence differences within Scandinavia. Cerebrovasc Dis 2002;14:161–168.
S115. Correia M, Magalhaes R, Silva MR, Matos I, Silva MC. Stroke types in rural and urban northern portugal: Incidence and 7-year survival in a community-based study. Cerebrovasc Dis Extra 2013;3:137–149.
S116. Feigin VL, Barker-Collo S, Parag V, et al. Auckland Stroke Outcomes Study. Part 1: Gender, stroke types, ethnicity, and functional outcomes 5 years poststroke. Neurology 2010;75:1597–1607.
S117. Vibo R, Korv J, Roose M. One-year outcome after first-ever stroke according to stroke subtype, severity, risk factors and pre-stroke treatment. A population-based study from Tartu, Estonia. Eur J Neurol 2007;14:435–439.
S118. Tentschert S, Parigger S, Dorda V, et al. Recurrent vascular events in patients with ischemic stroke/TIA and atrial fibrillation in relation to secondary prevention at hospital discharge. Wien Klin Wochenschr 2004;116:834–838.
S119. Eriksson M, Glader EL, Norrving B, et al. Sex differences in stroke care and outcome in the Swedish national quality register for stroke care. Stroke 2009;40:909–914.
