# Supplementary material for: Sex Differences in Disease Profiles, Management, and Outcomes Among People with Atrial Fibrillation After Ischemic Stroke: Aggregated and Individual Participant Data Meta-Analyses
Source: Womens Health Rep (New Rochelle). 2020 Jun 30;1(1):190–202. doi: 10.1089/whr.2020.0029 (PMC7784810; doi:10.1089/whr.2020.0029)
Supplement: Supplemental data [file Suppl_FigS2.pdf]

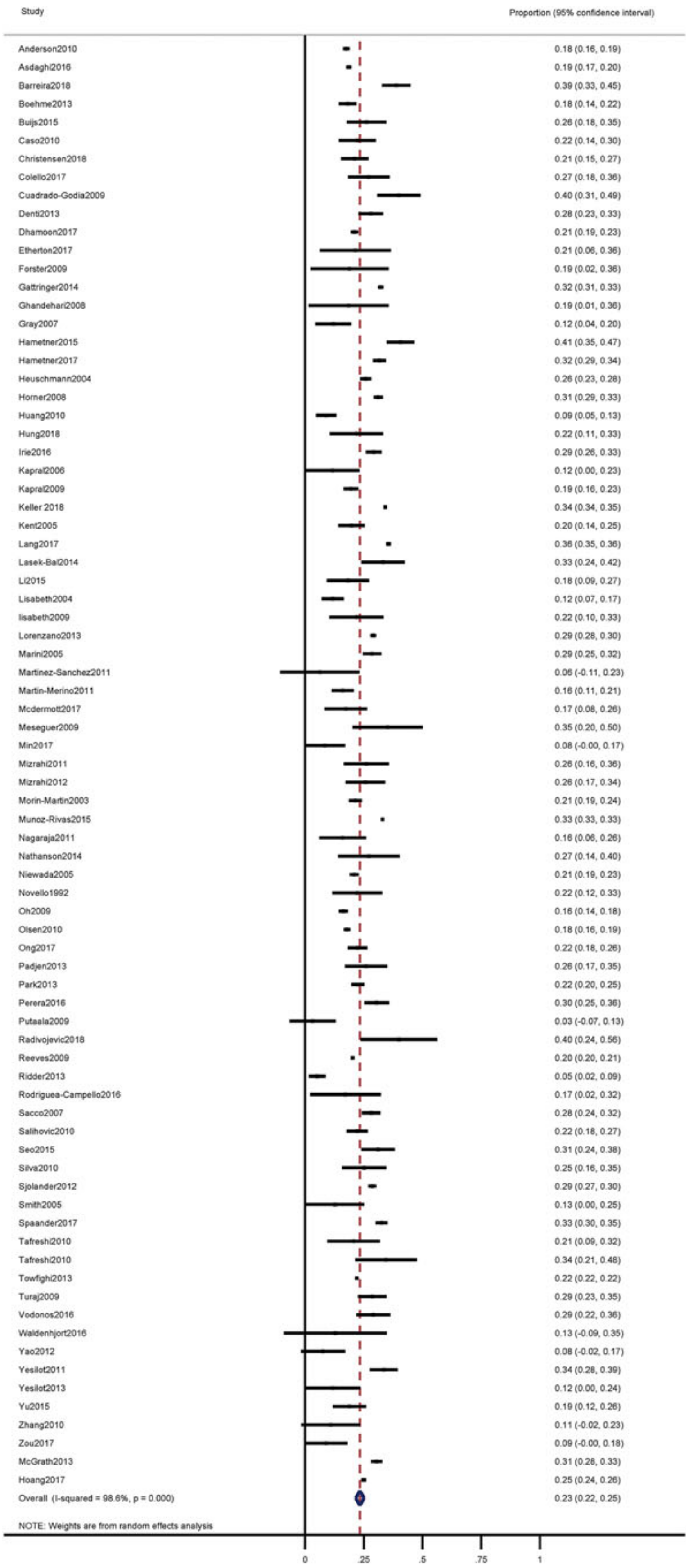

SUPPLEMENTARY FIG. S2. Proportion of AF in participants with ischemic stroke among women. A normal approximation was used to calculate the confidence interval, therefore, the negative lower confidence limits appear for anomalies. AF, atrial fibrillation.
