# Supplementary material for: Sex Differences in Disease Profiles, Management, and Outcomes Among People with Atrial Fibrillation After Ischemic Stroke: Aggregated and Individual Participant Data Meta-Analyses
Source: Womens Health Rep (New Rochelle). 2020 Jun 30;1(1):190–202. doi: 10.1089/whr.2020.0029 (PMC7784810; doi:10.1089/whr.2020.0029)
Supplement: Supplemental data [file Suppl_FigS7.pdf]

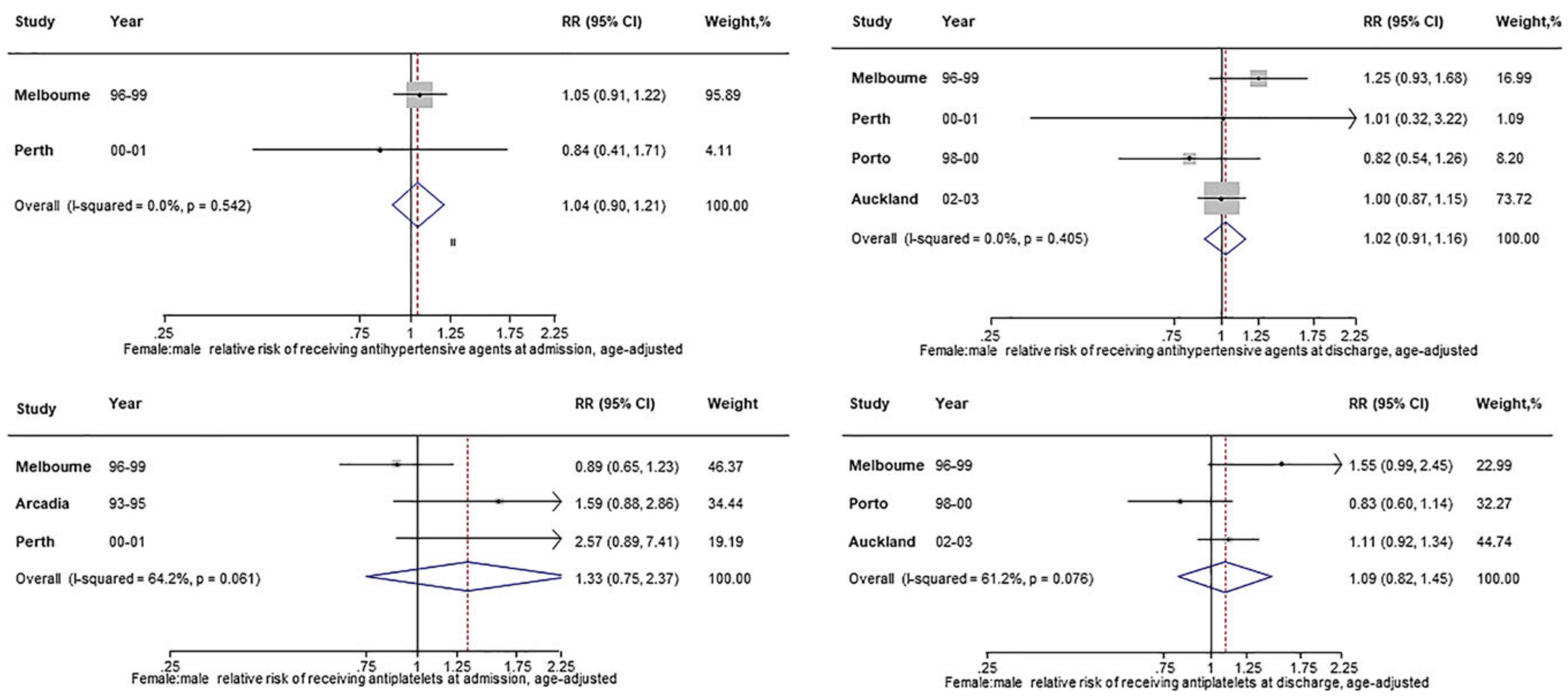

SUPPLEMENTARY FIG. S7. Sex differences in medication history among those with AF and ischemic stroke.
